# Supplementary material for: Hmong microbiome ANd Gout, Obesity, Vitamin C (HMANGO-C): A phase II clinical study protocol
Source: PLoS One. 2023 Feb 1;18(2):e0279830. doi: 10.1371/journal.pone.0279830 (PMC9891498; doi:10.1371/journal.pone.0279830)
Supplement: S1 File — (PDF) [file pone.0279830.s002.pdf]

**Protocol Title**	**H**mong **M**icrobiome **AN**d **G**out, **O**besity, Vitamin **C** (HMANGO-C)
**Principal Investigator/Faculty Advisor**	Name: Robert J Straka
	Department: College of Pharmacy
	Telephone Number: 510-900-9015
	Email Address: strak001@umn.edu
**Student Investigator**	Name: Ya-Feng Wen
	Current Academic Status (Student, Fellow, Resident): Student
	Department: Experimental and Clinical Pharmacology
	Telephone Number: 612-624-9683
	Institutional Email Address: wenxx164@umn.edu
**Scientific Assessment**	HRPP facilitated scientific assessment
**IND/IDE # (if applicable)**	N/A
**IND/IDE Holder**	N/A
**Investigational Drug Services # (if applicable)**	N/A
**Version Number/Date:**	Version 4/ November 19, 2021

PROTOCOL COVER PAGE

REVISION HISTORY

Revision #	Version Date	Summary of Changes	Consent Change?
2	6/6/2021	1. Add COVID-19 vaccine questions to the questionnaire. Previously, we only ask the COVID-19 positive test. With many people received the COVID-19 vaccine, it is important for the study to collect the vaccination status of the participants.<br>2. Clarify the consent language (both English and Hmong) under "What will be done with my data and specimens when this study is over?" so the participants can better understand how the investigators handle the data and specimens.	Yes
3	9/23/2021	1. Revise the definition of hyperuricemia from 6 mg/dL to 6.8 mg/dL based on the 2020 American College of Rheumatology Guideline for the Management of Gout<br>2. Elaborate the “Word of Mouth” recruitment strategies to include incentives in the recruitment section.<br>3. Revise the study process regarding the collection of medication history, changing “will” to “may” in the following sentence: Participants “may” be asked to bring their medications with them to the enrollment visit and “may” be asked about their adherence to these medicines.<br>4. Update the study flyers in both English and Hmong to include study website and locations where we intend to recruit the participant from.<br>5. Replace the currently used dietary survey to DHQ-3 dietary survey. (DHQ-3 Dietary History Questionnaire is a web-based survey. Therefore, the paper-based DHQ-1, which is similar to DHQ-3, is submitted for review.)	No
4	11/19/2021	1. Revise the consent form on the risk of blood draw. Change the language from “When blood is taken, sometimes people feel a little faint or dizzy for a short time” to, “When blood is taken, sometimes people may faint, feel dizzy or off balance for a short time”. The consent form in Hmong does not have the word "feel a little” so the most recent consent form in Hmong has the same meaning as the revised consent form in English.<br>2. Remove the exclusion criteria of “Serum urate measured at enrollment visit > 10 mg/dL”, because it is important to include individuals who are at a higher risk of developing gout and study the effect of vitamin C in this population.	Yes

Table of Contents

1.0 Objectives ... 5
2.0 Background ... 5
3.0 Study Endpoints/Events/Outcomes ... 8
4.0 Study Intervention(s)/Investigational Agent(s) ... 8
5.0 Procedures Involved ... 9
6.0 Data and Specimen Banking ... 12
7.0 Sharing of Results with Participants ... 14
8.0 Study Population ... 16
10.0 Local Number of Participants ... 19
11.0 Local Recruitment Methods ... 19
12.0 Withdrawal of Participants ... 22
13.0 Risks to Participants ... 22
14.0 Potential Benefits to Participants ... 24
15.0 Statistical Considerations ... 24
16.0 Health Information and Privacy Compliance ... 26
17.0 Confidentiality ... 28
18.0 Provisions to Monitor the Data to Ensure the Safety of Participants ... 29
19.0 Provisions to Protect the Privacy Interests of Participants ... 31
20.0 Compensation for Research-Related Injury ... 32
21.0 Consent Process ... 32
22.0 Setting ... 33
23.0 Multi-Site Research ... 34
24.0 Coordinating Center Research ... 34
25.0 Resources Available ... 34
26.0 References ... 36

ABBREVIATIONS/DEFINITIONS

- ARDL: Advanced Research and Diagnostic Laboratory
- COVID-19: Coronavirus disease 2019
- CUHCC: Community-University Health Care Center
- DOT: Department of Transportation
- GAQ2.0: Gout Assessment Questionnaire 2.0
- HU: Hyperuricemia
- MCC: Minnesota Community Care
- OHSA: Occupational Safety and Health Administration
- PPE: Personal protective equipment
- SARS-CoV-2: The virus that causes COVID-19
- SU: Serum Urate
- T2DM: Type 2 Diabetes
- ULT: Urate-Lowering Therapy
- UMGC: University of Minnesota Genomics Center
- UMN: University of Minnesota

1.0 Objectives

The objectives of this study include:

- Quantify the impact of vitamin C on patient outcomes, including serum urate level, gout-related symptoms, and obesity (measured by BMI) in both healthy Hmong adults and in Hmong patients with hyperuricemia (HU) and/or gout
- Identify associations between individuals' taxonomic and functional patterns of gut microbiota and its impact on the serum urate lowering effect of vitamin C
- Compare taxonomic and functional patterns of gut microbiota between people with HU and/or gout and people without HU and gout
- Identify associations between individuals' taxonomic and functional patterns of gut microbiota and self-reported acute gout trigger foods

Study design is depicted in **Figure 1**.

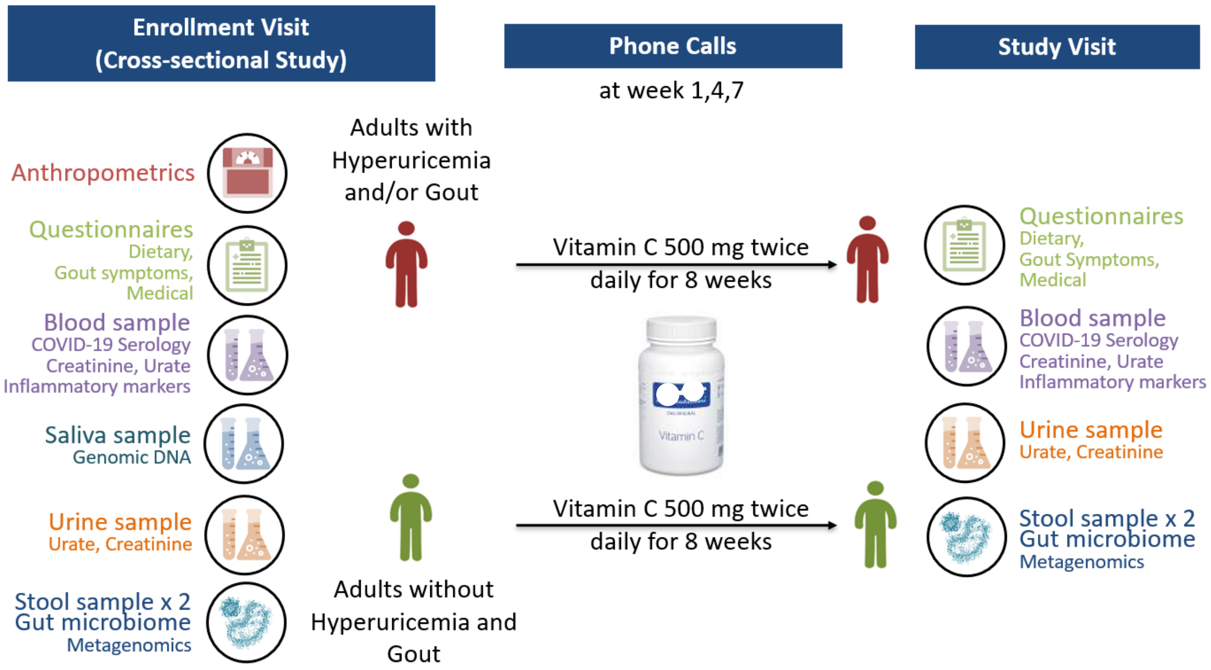

Figure 1. Study Design

2.0 Background

Gout, caused by chronic elevation of serum urate (SU), is the most common form of inflammatory arthritis worldwide.[1] About 3.9% of adults in the U.S. suffer from gout[2] and prevalence is even higher in certain ethnicities. Factors that may influence SU include patients' characteristics (gender, weight, renal function, etc.), genetics, and diet. Foods and beverages that have shown positive association with HU and gout are alcohol (particularly beer), purine-rich foods, red meat, seafood, and sugar-sweetened drinks, while inverse association has been found with dairy intake such as skimmed milk and low-calorie yoghurt, coffee and vitamin C.[1]

The gut microbiota composition and function have been linked to common chronic human disorders, such as obesity, diabetes, non-alcoholic fatty liver disease, and rheumatoid arthritis.[3,4] Intestinal microbiota of gout patients were also found highly distinct from healthy individuals in both organismal and functional structures from a small study conducted in China.[5] Strategies to use personal microbiome features to predict glucose response to specific food have been proposed.[6] However, **little is known about the impact of microbiota on food and urate-lowering therapy (ULT)**. The translational significance of a microbiota-guided approach to select appropriate foods and medications that could prevent the elevation of SU for individuals with gout or at high risk for gout is significant.

The Hmong are a unique Asian sub-population. Hmong men exhibit a 2-fold higher prevalence of gout, manifesting it earlier in life and experiencing up to 5-fold increased risk of gout-associated complications, compared to non-Hmong in the US.[7,8] This could lead to higher rates of cardiometabolic diseases (e.g. hypertension, renal disease, type 2 diabetes [T2DM]), which significantly impact morbidity, mortality and healthcare costs.[9,10] To prevent acute gout attack and reduce complications associated with gout and/or HU, it is critical to maintain UA below 6 mg/dL.[11-13]

In this study, we will test four hypotheses and thereby accomplish our objectives as following:

Ho (1): High dose vitamin C intervention improves outcomes in (a) healthy adults and (b) patients with HU and/or gout.

Rationale: Previous studies had demonstrated vitamin C 500 mg twice daily for 8 weeks is effective in lowering serum urate compared to placebo in healthy adults.[14] However, one study in patients with gout receiving vitamin 500 mg once daily for 8 weeks did not find significant serum urate reduction in patients on allopurinol.[15]

We will test this Ho1 (a) and (b) by giving vitamin C 500 mg twice daily to all the study participants and analyze by 3 groups: 1) participants with HU and/or gout with ULT, 2) participants with HU and/or gout without ULT, and 3) participants without HU and gout.

The outcome measures include both biological surrogate marker (serum urate), patient reported outcome (gout-related symptoms), and obesity (measured by BMI).

Ho (2): The urate-lowering effect by vitamin C is influenced by gut microbiome.

Rationale: Humans cannot produce their own vitamin C; therefore, dietary is an important source of vitamin C. However, recent computational analysis identified *Burkholderia*, *Pseudomonas* and *Erwinia* possessed the machinery to produce ascorbate. These genera are commonly seen in patients with Crohn's disease. The presence of these organisms can impact the production of the anti-inflammatory ascorbate metabolite. This could have an impact on the treatment effect (most likely the anti-inflammatory effect) of vitamin C in patients with gout.

A few variables including genetic variants and viral infections (such as SARS-CoV-2 infection) could confound the association between the urate-lowering effect by vitamin C and gut microbiome. Genetic variants such as rs2231142G>T within *ABCG2* has shown to be associated with reduced SU-lowering response to allopurinol, a commonly used ULT, within patient populations primarily comprised of Caucasians [16-18]. We also identified an alternate variant within *SLC22A12* (rs505802C>T) that was statistically associated with reduced SU-lowering response to allopurinol *and* a two-fold lower drug exposure in Hmong population.[19] Urate-lowering effect vitamin C may also be impacted by the similar or other novel genetic variants. Increased SU to promote helper T cells also has been observed in mouse model infected with respiratory syncytial virus (RSV), the most common causes of bronchiolitis and pneumonia in infants.[20] SARS-CoV-2, the 2019 novel coronavirus, has infected more than 18 millions people in the US as of December 2020, primarily in elderly and individuals with comorbidities. There is currently lack of evidence of how SARS-CoV-2 can affect SU. However, the activation of immune system causing systematic inflammation is a common complication in patients with SARS-CoV-2. [21,22] The SARS-CoV-2 infected individuals may have increased SU as a result.

Therefore, we will test for genetic variants that have associations with HU, gout, the responses of ULTs, and medications that participants may be taking during the study. Also, we will test the serology for SARS-CoV-2 infection.

We will test this Ho2 by analyzing the urate-lowering effect of vitamin C with the consideration of the composition of gut microbiome.

Ho (3): Taxonomic and functional patterns of gut microbiota are associated with HU and/or gout.

Rationale: Certain taxonomic and functional patterns of gut microbiota are associated with increased bioavailability of purines, which increases SU. Individuals with a high abundance of hypoxanthine producing flora in the gut and a low abundance of hypoxanthine consuming flora in the gut could be at risk of higher hypoxanthine absorption, which could translate to higher SU. High hypoxanthine consumers in the microbiota could result in increased uric acid in the colon, but we have not seen colonic urate correlated with SU in a recent inflammatory bowel syndrome cohort.

We will test this Ho3 by comparing the taxonomic and functional patterns of gut microbiota of those with HU/gout (with or without ULT) to those without HU or gout.

Ho (4): Food types that trigger gout can be predicted by individuals' taxonomic and functional patterns of gut microbiota.

Rationale: There is a strong correlation between an individual's taxonomic patterns of gut microbiota and self-reported trigger foods in patients with type 2 diabetes.[6] The correlation could also be present in patients with HU and gout.

We will test this Ho4 by comparing the taxonomic patterns of gut microbiota of individuals with gout who have similar self-identified trigger foods to those who have different trigger foods.

3.0 Study Endpoints/Events/Outcomes

3.1 Primary Endpoint/Event/Outcome: Absolute change in serum urate after the 8 week of vitamin C intervention

3.2 Secondary Endpoint(s)/Event(s)/Outcome(s): Secondary outcomes include the followings:

- Change in gout related symptoms measured by Gout Assessment Questionnaire 2.0[15] and number of joints affected by gout using Swollen Joint Count (44 Joints) post-administration of vitamin C
- Change in BMI
- Taxonomic and functional patterns of gut microbiota associated with SU
- Change in taxonomic and functional patterns of gut microbiota post-administration of vitamin C
- Taxonomic and functional patterns of gut microbiota associated with food types that trigger gout

4.0 Study Intervention(s)/Investigational Agent(s)

4.1 Description:

All participants will be taking vitamin C 500 mg twice daily by mouth for 8 weeks. All participants will continue taking their prescribed medications during the study period.

Vitamin C is commercially available over-the-counter food supplements. Vitamin C will be purchased from a GMP manufacturer (such as Pure Encapsulations, Sudbury, MA) which can demonstrate a good quality and safety of the product.

4.2 Drug/Device Handling:

The vitamin C will be stored securely in a locked cabinet at Dr. Straka's office. Vitamin C is an over-the-counter product and will be distributed by research staff as per applicable regulations. The label will list the name of the study, the number and frequency of tablets to be taken, the participant's name and ID number, and the phone number for a pharmacy helpline. A study dispensing and accountability log will be maintained by bilingual research staff. The participants will be reminded to return the study pill containers and any remaining tablets at Week 8.

4.3 Biosafety: This study does NOT involve recombinant or synthetic nucleic acid molecules (r/sNA; RNA, DNA), infectious agents, or biologically-derived toxins.

4.4 Stem Cells: This study does NOT involve embryos or embryonic stem cells.

4.5 Fetal Tissue: This study does NOT involve human fetal tissue or cell lines derived from human fetal tissue.

5.0 Procedures Involved

5.1 Study Design: This is an open-label interventional study. We aim to enroll 2:1 matching between participants with HU and/ or gout and participants without HU and gout. We aim to have 3 analysis groups of about 60 people per group (total about 180 participants): 1) people with HU or gout with ULT, 2) people with HU or gout without ULT, 3) people without HU or gout without ULT.

5.2 Study Procedures:

Recruitment process: Our recruitment and enrollment plan in the Minneapolis/St. Paul MN metropolitan area includes outreach, electronic, print, and social media to ensure we can enroll 180 participants to reach statistical power. (*Recruitment flyers and scripts uploaded as supplements.*) Depending on COVID-19 restrictions during enrollment, we are planning on collecting data either together in person with a research staff (which can happen at a clinic, another location of their choice, or at their home), or separately with the participants collecting data in their home, with research staff supporting them remotely by phone or by Zoom, as participants prefer.

Enrollment visit (Visit 1): The enrollment visit will take approximately 120 minutes. During the visit, bilingual (English and Hmong) researchers will review and discuss the study, consent form, and HIPAA form in participants' preferred language. Any questions will be answered, and the researcher will assess if potential participants understand what is expected of them before they sign the consent and HIPAA forms.

Once the consent form is signed, participants will complete questionnaires about their medical history, gout symptoms, lifestyle activities (including diet and tobacco, and exercise) and medication history (including prescription, over-the-counter medications, probiotics and herbal therapies). Researchers will measure anthropometry (height, weight, and waist- circumference), and vital signs (temperature, respiratory rate, oxygen saturation, blood pressure and heart rate).

Trigger-food profiling: Researchers will ask participants to identify their gout flare trigger foods, first by an open-ended question and then by reviewing a list of common trigger foods, developed with a Hmong registered dietician. We will ask if there is any known "amount" (dose) of the trigger food known to them and have them grade them as the "highest, moderate or lowest" association with triggering gout in them.

Medication history: Participants may be asked to bring their medications with them to the enrollment visit and may be asked about their adherence to these medicines. If

necessary, the researchers will ask for permission to get a current medication list from their medical clinic.

Gout Symptoms: The modified Gout Assessment Questionnaire 2.0 [23] (modified by a cultural equivalent approach) and Swollen Joint Count (44 Joints) (https://www.carearthritis.com/tools/tools_html.anonlaunch?toolid=8&refid=/physicians.php%23tab2) will be used.

Stool collection: Participants will be instructed in stool collection and will be given stool collection kits to take home with them for 2 consecutive days. They will return the samples at their convenience. Upon receipt at the study lab, the samples will be stored at –80°C until DNA extraction.

Blood collection: We will be offering two methods: 1) in person and 2) at home method. In person blood draws will be performed by a phlebotomist available at the visit and later for subjects who are not ready to consent at the visit but choose to consent later. Blood samples will be collected into serum collection tubes (BD 366668-1 Vacutainer® Plus Plastic Serum Blood Collection Tubes or equivalent) and stored at -20°C until testing. For at home blood collection, capillary blood collection tubes (Microvette® CB 200 uL or equivalent) will be available to participants for self-collection. After self-collection, participants will ship samples to Straka's lab according to the regulation of Occupational Safety and Health Administration (OHSA) and Department of Transportation (DOT) to and stored at -20°C until testing.

We will test for serum urate and creatinine to assess kidney function (calculate eGFR), which is a confounding contributor to SU. Inflammatory markers link to gout and obesity will also be tested.

Viral serology (antibody test) related to SARS-CoV-2 will be tested using the method developed and validated at the Advanced Research and Diagnostic Laboratory (ARDL), University of Minnesota.

Saliva collection: DNA will be extracted from the saliva samples collected using a commercial collection kit. Genetic variants that are associated with HU, gout, the responses of ULT, or other medications which the participants may be taking during the study will be tested. Although others may be examined as this field of study evolves, some known examples of genes that will be assessed for genetic variations that impact either medications used or the conditions of HU and/or gout include *ABCG2, and SLC22A12.*[24]

Urine collection: Participants will be asked to collect one urine sample during the visit. Urine urate and creatinine will be measured. Urine samples will be stored for additional analyses as needed.

During the 8 Week of Study: The bilingual (English and Hmong) researchers will contact each participant by phone (either text or phone call as participants desire) at **weeks 1, 4, and 7** during the study to assess any medication safety and tolerance issues, any

medication changes, and remind participants of the importance of precise timing of dosing leading up to final visit.

Study Visit (Visit 2): All the information collected in Visit 1 will be collected again at this visit except for informed consent, privacy statement, DNA and demographics. This visit will last approximately 120 minutes.

Table 1. Clinical data collected in each study visit

Items	Visit 1	4 Phone calls*	Visit 2	Site**
Informed consent and privacy statement (HIPAA)	X			A/B
Demographics	X			A/B
Anthropometrics: height, weight, waist circumference	X		X	A/B
Medical history, including pregnancy status and COVID-19 vaccine status	X		X	A/B
Medication and dietary history	X		X	A/B
Vital signs (BP, HR)	X		X	A/B
Gout symptoms (GAQ2.0 and swollen joint count)	X		X	A/B
Blood, saliva and urine samples collection	X		X	A/B
Stool samples collection x 2 consecutive days	X		X	B
Medication adherence		X	X	A/B
Adverse effects from study medication		X	X	A/B
Blood samples: urate, creatinine, SARS-CoV-2 IgG analysis	X			C
Saliva samples: DNA extraction and genotyping				D
Urine samples: uric acid and creatinine analysis	X		X	C
Stool sample: preparation and sequencing				D
Length of visit	120 mins	5-10 mins	120 mins	

* Four phone calls will be conducted at the end of week 1, 4, and 7.
**Sites: (Locations of various activities/procedures)

1) A = Community-University Health Care Center (CUHCC-A1), the primary participating clinic site and/or Minnesota Community Care (MCC-A2) a secondary participating clinic/site.
2) B = Participants' home
3) C = Advanced Research and Diagnostic Laboratory (ARDL), University of Minnesota
4) D = University of Minnesota Genomics Center (UMGC) for genotyping samples

NOTE: In response to reducing risk for COVID-19 infection for study participants, all the activities conducted at A locations (the participating clinic sites) will also be offered as an option for no in-person contact at participants' home (B locations). Methods include phone/online interview (using REDCap to interview participants) or a phone/online hybrid (participant fills out questionnaires on their own using internet access or paper copies and recruiter/interviewer would be on phone for assistance). The collection of blood, saliva, urine, and stool will be performed by participants at their home. Once the samples have been collected, participants will contact research staff and research staff will go to participants' homes with personal protective equipment (PPE) including masks, shields, gloves, and gowns to collect the samples. During the collection process, support from research staff will be provided if needed through phone or Zoom per participants' preference.

(*Instrumental questionnaires, including demographic, medical, medication, dietary, gout symptoms questionnaires were uploaded as supplements.*)

The data repository center and clinical coordinating center will be located in Dr. Knights' laboratory in University of Minnesota (UMN). Dr. Knights and co-investigators (specifically Drs. Robert Straka and Kathleen Culhane-Pera) will review accrual, drop-outs, and any protocol deviations on a periodic basis (monthly or weekly depending on the criteria developed in our planned detailed monitoring schedule).

5.3 Study Duration:

- The duration anticipated for an individual participant's participation in the study is 8 weeks.
- It is anticipated that recruitment and intervention would take between 6 and 12 months.
- Laboratory and data analysis are anticipated to be completed within 2 years.

5.4 Use of radiation*:* This research does NOT involve the use of radiation.

5.5 Use of Center for Magnetic Resonance Research*:* This research will NOT involve the Center for Magnetic Resonance Research facilities.

6.0 Data and Specimen Banking

6.1 Storage and Access:

The study consent form provides participants the **option** to agree for use of their samples in future research. For those participants who agree, any stool, urine, or blood samples remaining after analysis is complete will be stored in secured UMN -80 freezers for future studies.

Identifiers will be available only to the research team throughout the duration of the data collection period, in order to contact participants for safety and

adherence. At the end of the data collection period, the map between participants' identifiers and their unique study ID will be maintained on a full-disk encrypted secure server behind the UMN firewall, with access only by the PI and co-Is. Identifiers will only be accessed again at the time of the dissemination event, when invitations to the participants and the broader community will be sent out to share the results of the study.

Once data is entered into the database, hard copy (paper versions of) surveys will be stored in a locked file cabinet of the PI's locked office in a secure UMN building. These paper surveys will be maintained until the results of the study are published, at which point they will be securely disposed.

The digital data for this study will be entered into a REDCap database, which uses a MySQL database via a secure web interface with data checks used during data entry to ensure data quality. REDCap includes a complete suite of features to support HIPAA compliance, including a full audit trail, user-based privileges, and integration with the institutional LDAP server. The MySQL database and the web server will both be housed on secure servers operated by the University of Minnesota Academic Health Center's Information Systems group (AHC-IS). The servers are in a physically secure location on campus and are backed up nightly, with the backups stored in accordance with the AHC-IS retention schedule. Backup tapes are stored offsite. The AHC-IS servers provide a stable, secure, well-maintained, and high-capacity data storage environment, and both REDCap and MySQL are widely-used, powerful, reliable, well-supported systems. Access to the study's data in REDCap is restricted to the members of the study team by username and password.

Participant accrual data will also be entered into OnCore as required by the University of Minnesota's Academic Health Center. OnCore is a suite of clinical and translational research modules consisting of software for research, patient registry, and biospecimen management.

The AHC IE will not be used for data and BioNet will not be used for any specimens.

6.2 Data:

Information about participants' demographic, anthropometrics, medical conditions, medication use, and dietary patterns will be collected, in addition to stool, urine, and blood samples. Participant samples will be stored and analyzed by unique study identification numbers only. Only the study team has the link between samples and individual participants and linkage will not be released outside of this trial. Study data will be presented and published only in ways that make it impossible to identify individual participants.

6.3 Release/Sharing:

For participants who agreed to have their samples used for future research, these samples may be shared for that purpose with outside collaborators at the investigator's discretion, with input from Hmong Gout Coalition members, only after appropriate regulatory approval has been given, and a material transfer agreement is in place.

7.0 Sharing of Results with Participants

7.1 Participants will receive individual results including vital signs, anthropometry, renal function, serum urate level, COVID19 antibodies, genetics, and microbiome results. We will also share the aggregated genetics and microbiome results with participants. However, we will stress that analysis of biological samples is for research purposes only and are not necessary for participants' clinical care.

7.2 Sharing of genetic testing:

7.2.1 Disclosure of results:

Selected individual and aggregated genetic testing results will be returned to participants. We are only analyzing specific genes related to HU, gout and medicines commonly used to treat gout. Although genes related to response to other medications may be examined, we are not testing for genes associated with other diseases, for example those associated with risks for cancer, seizures, or psychiatric diseases; we will not report any incidental findings, including genetic relationships (relatedness) between people. In addition, genetic testing will be conducted in a research lab instead of in a lab certified under Clinical Laboratory Improvement Amendments (CLIA) and consequently we are recommending any result be not considered for interpretation in a clinical situation.

7.2.2 If returning results to participants:

- Aggregate or individual results:
  Participants will receive both individual and aggregated results. Aggregate results will be presented in a way that does not allow participants to see other participants' genotypes.
- Laboratory results:
  The genetic testing will be conducted in a research laboratory instead of a lab certified under Clinical Laboratory Improvement Amendments (CLIA). Participants will be informed that the results are for research use only and should not be used for routine clinical care. Contact information for a CLIA laboratory will be provided (such as UMMC Molecular Diagnostic Laboratory) if the participants choose to confirm the results. However, participants will have to pay for this clinical information.
- Plan for return of results to participants:

Aggregated results will be shared at the conclusion of the study with the participants and the Hmong community at several venues and through several media. These may include locations, such as recruitment sites, community conferences, community events, community organizations and media including Hmong radio and television, and the Hmong Gout Coalition website. In addition, we will share results with local physicians and pharmacists who have partnered with recruitment, and we will disseminate our results with broader audiences by presenting at local, national, and international conferences, and by publishing peer-reviewed articles in scientific journals.

Individual participant results will also be shared at the conclusion of the study. We will mail relevant individual results to the participants. Since the genetic results will not affect routine clinical care, credentialed genetic professionals will not be used to explain study results in-person.

- Types of results to be returned to participants:
  a. Personal anthropometry and vital signs
  b. Personal blood test results: SU before and after Vitamin C
  c. Personal microbiome results: Summary of microbiome results, before and after vitamin C, and any correlations with trigger foods
  d. Personal genetic results**: We will only be returning genetic variants on genes which have been shown to be associated with urate absorption and reuptake, including *ABCG2* and *SLC22A12*.
  e. Personal SARS-CoV-2 antibody test results

**There is still a lack of evidence or consensus supporting the clinical interpretation of these genetic variants and microbiomes and their impact on HU/gout and or response to drug therapy. This therefore limits any important interpretation. However, since the Hmong community has consistently expressed their desire to receive information on their results, we have agreed to provide our research findings or select data above, noting they are of limited, if of any, clinical value. There will be no concerns with incidental findings because we are only looking for genetic variants that are associated with the treatment efficacy and not disease-causing genetic variations.

7.2.3 Future analysis of genotypes:

The research team will offer the participants options for future use of their de-identified DNA material, which they will indicate on their consent form, including

their permission for: our research team or other research teams to analyze their DNA for any future research study; or our research team or other research teams to analyze their DNA only for future research studies about genes and medicines without contacting them; or their permission for us to contact them about possible future studies.

For participants consenting for further analysis of the significance of genotypes in the future, the research team may conduct additional studies to examine the impact of genetic variants on the risk of HU and gout or other related research questions, with IRB permission. In such cases, additional IRB approval will be sought.

8.0 Study Population

8.1 Inclusion Criteria:

- Self-identified Hmong men and women whose both parents are Hmong, ≥ 18 years of age, with and without hyperuricemia (serum urate ≥ 6.8 mg/dL) and/or gout (defined by 2020 Gout Classification Criteria defined by American College of Rheumatology)[25] are eligible for the study
- Willing and able to provide informed consent
- Willing and able to adhere to the study protocol

Gout group: Individuals with hyperuricemia and/or gout are defined as:

- serum UA ≥ 6.8mg/dL based on the baseline measurement (with or without stable ULT*)
- serum UA < 6.8 mg/dL based on the baseline measurement (with or without ULT) with at least 1 episode of peripheral joint or bursal swelling, pain, or tenderness (acute gout flare) in their lifetime

*ULT= Urate Lowering Therapy, includes allopurinol, febuxostat, probenecid, lesinurad, and benzbromarone

Non-gout group: Individuals without gout, without ULT, and with serum UA <6.8 mg/dL. Individuals who are siblings or other family members to those with gout are allowed

8.2 Exclusion Criteria:

Participants with following conditions will be excluded from the study:

- Allergy or sensitivity to vitamin C
- Diagnosis/history of:
    - Gastrointestinal surgery including colectomy, ileectomy, and gastrectomy
    - Inflammatory bowel disease

    - Auto-immune disease
    - Type I diabetes mellitus
    - Severe kidney disease (i.e., on dialysis)
    - End-stage liver disease (i.e. cirrhosis)
    - Pregnant women
    - Breastfeeding women
    - Glucose-6-phosphate dehydrogenase deficiency (due to increased bleeding risk in patients with G6PD deficiency when receiving vitamin C)
- Current use of:
    - Antibiotics
    - Probiotics supplement
    - Ketogenic diet

8.3 Screening:

We will use specific selection criteria to narrow the recruitment pool as much as possible to those potentially eligible. Participants who are interested in participating will be administered an eligibility assessment over the telephone prior to enrollment. Assessments resulting in questionable eligibility will be reviewed by the study physician for a final determination prior to enrollment.

9.0 Vulnerable Populations

9.1 Vulnerable Populations:

Population / Group	Identify whether any of the following populations will be targeted, included (not necessarily targeted) or excluded from participation in the study.
Children	Excluded from Participation
Pregnant women/fetuses/neonates	Excluded from Participation
Prisoners	Excluded from Participation
Adults lacking capacity to consent and/or adults with diminished capacity to consent, including, but not limited to, those with acute medical conditions, psychiatric disorders, neurologic disorders, developmental disorders, and behavioral disorders	Excluded from Participation
Non-English speakers	Included/Allowed to Participate

Those unable to read (illiterate)	Excluded from Participation
Employees of the researcher	Included/Allowed to Participate
Students of the researcher	Included/Allowed to Participate
Undervalued or disenfranchised social group	Excluded from Participation
Active members of the military (service members), DoD personnel (including civilian employees)	Included/Allowed to Participate
Individual or group that is approached for participation in research during a stressful situation such as emergency room setting, childbirth (labor), etc.	Excluded from Participation
Individual or group that is disadvantaged in the distribution of social goods and services such as income, housing, or healthcare.	Included/Allowed to Participate
Individual or group with a serious health condition for which there are no satisfactory standard treatments.	Excluded from Participation
Individual or group with a fear of negative consequences for not participating in the research (e.g. institutionalization, deportation, disclosure of stigmatizing behavior).	Excluded from Participation
Any other circumstance/dynamic that could increase vulnerability to coercion or exploitation that might influence consent to research or decision to continue in research.	Excluded from Participation

9.2 Additional Safeguards:

Some people in our recruitment population will have characteristics of vulnerable populations, including: low literacy, non-English speaking, which increase the risks of their not fully understanding the research process and limits their ability to provide full informed consent, particularly in a complex study design. We have successfully recruited and enrolled people with these characteristics in the Hmong community before. By working closely with Hmong

Gout Coalition members and bilingual/bicultural research team members, the team has explored the meanings of heredity, genetics, and gene testing to identify how to translate these scientific concepts into cultural understandings; has created written explanations and consent processes in fifth-grade English and visual representations of the research goals and processes (such as info-graphics); has translated these into Hmong language that capture the meanings without being word-for-word translations that could interfere with people's understandings and have had these translations reviewed by another bilingual/bicultural researcher who is trained in research ethics; and has carefully chosen verbal explanations of gout, gout treatments, and research about the relevance of genetics and gout treatment in English and Hmong, so that the messages are clear. These language adaptations ensure that all bilingual/bicultural research team members are able to connect with people whom they are recruiting and consenting. In addition, we have responded to UMN IRB requirements that we consider their Hmong template in creating our consent form, use their short form for non-English speaking participants, attest to our translation processes, and provide contact information for a Hmong speaker outside of the study team who understands the research process, for anyone in the study who wants to speak in Hmong about the research processes.

10.0 Local Number of Participants

10.1 Local Number of Participants to be Consented:

We plan to consent 180 participants in the Minneapolis/St. Paul MN metropolitan area.

11.0 Local Recruitment Methods

11.1 Recruitment Process: Our recruitment and enrollment plan in the Minneapolis/St. Paul MN metropolitan area is broad and deep to ensure we can enroll sufficient participants to reach statistical power. (**Table 1**) Our recruitment approaches with the Hmong Gout Coalition (HGC) members will include connections with local Hmong organizations and events; messages on media frequented by Hmong community members; and possibly, if needed, collaborations with Hmong healthcare professionals working in Hmong pharmacies and primary care clinics with Hmong patients. In addition, we can recruit through the University of Minnesota Study Finder website (https://studyfinder.umn.edu/) and Study Finder kiosks available on the University of Minnesota campus.

Table 1 Recruitment Plans Summary

Type	Organizations/Institute	Processes
Social media	• Facebook: Hmong Gout Coalition (HGC) https://www.facebook.com/	1. Add information to HCG Facebook and website

	HmongGoutCoalition/ and Hmong Gout Pain and Health<br>• HGC website https://www.hmonggoutcoalition.org<br>• YouTube channels hosted by HGC	2. Encourage others to "like" and "share" with others suffering with gout<br>3. A live Q&A session explaining the study in both English and Hmong on YouTube channels hosted by HGC
Word of mouth<br>- snowball	• People enrolled in previous studies and provided consent to recontact for future studies (GOUT-H, VIP Hmong Study)<br>• Family members of enrollees who have enrolled<br>• Famous people with gout	1. Research staff will reach out to the participants from previous studies<br>2. Ask enrollees to pass information to other people with gout. Enrollees will not provide potential participants' contact information to the study team. The potential participants can decide whether they want to reach out to the study team or not.<br>3. Enrollees will be provided with $10 USD up to $30 USD when the potential participants who are referred by the study enrollees contact the study team. The payment will not be contingent on whether the potential new participants enroll to the study or not.
Local Hmong organizations	• 18 Clan Council and their family councils<br>• Hmong college study associations<br>• Hmong churches<br>• Hmong HealthCare Professions Coalition (HHCPC)	Partner with the organizations to:<br>1. Meet their members at their usual meetings and locations to talk about the study and gout<br>2. At the same meeting, enroll people who meet criteria, or return at later date to enroll<br>3. Invite people to call phone numbers to learn more and schedule an enrollment date
Media	• Hmong Radio<br>• Hmong TVs<br>• Hmong newspapers	Partner with owners of media/ programs to:<br>1. Have well-known people with gout present with Hmong physicians and pharmacists to educate about gout and advertise the study<br>2. Invite people to attend enrollment locations at specific times and dates

		3. Invite people to call phone numbers to learn more and schedule an enrollment date
Academic Institutions	• University of Minnesota Study Finder website (https://studyfinder.umn.edu/)<br>• Study Finder kiosks available on the University of Minnesota campus.	1. Once the study is approved by IRB, we will register the study on Study Finder website allowing students, employees, and general public to involve in the study
Pharmacies	• Hmong owned pharmacies:<br>• Phalen Family Pharmacy<br>• Como Pharmacy<br>• Brightly Pharmacy<br>• General pharmacies with Hmong staff, Walgreens, CVS/Target, Cub, etc.	At each pharmacy site, general recruitment in Hmong and English:<br>1. Pharmacists can highlight the research project information to Hmong patients with gout<br>2. Flyers about study in lobbies and exam rooms<br>3. Posters about gout education and research<br>4. Videotapes about gout education
Medical clinics (primary care and specialty- Rheumatology and Urology)	• Hmong Medical Association (HMA)<br>• Minnesota Community Care (Primary contact: Dr. Muaj Lo)<br>• HealthPartners (Primary contact: Dr. Steve Mouacheupao)<br>• Medica/ Allina (Primary contacts: Drs. Mai See Moua and Chaneng Vang)<br>• Independent clinics (Primary contacts: Drs. Phua Xiong and Peter Yang)<br>• Bethesda Family Physicians- UMN Clinic (Dr. Ann Philbrik)	At each clinic site, targeted recruitment in Hmong and English:<br>1. Clinicians with permission and appropriate access will search their patient EMR base by criteria<br>2. Clinics will send recruitment letters with their clinician's name as a supportive partner<br>3. Interested people call and talk with Hmong bilingual/ bicultural research staff to discuss the study on our dedicated phone line and be invited to an enrollment session<br><br>At each clinic site, general recruitment in Hmong and English:<br>1. Flyers about study in lobbies and exam rooms<br>2. Posters about gout education and research<br>3. Videotapes about gout education videotapes

11.2 Identification of Potential Participants: The methods to identify potential participants are listed in section *11.1*. We will not utilize a Clinical Electronic

database (such as Academic Health Center Information Exchange, AHC-IE) to identify potential participants.

11.3 Recruitment Materials: Recruitment flyer and video recording will be used to recruit participants.

11.4 Payment: Participants will be paid $75 for the completion of the first visit (after receiving a stool sample from the participants) by mailing the Greenphire ClinCard to participants. We will deposit another $75 for the completion of the second visit (after receiving a stool sample from the participants) to the same Greenphire ClinCard they have received in the first visit.

12.0 Withdrawal of Participants

12.1 Withdrawal Circumstances: If the health or welfare of a participant is in question, for any reason, and cannot be ameliorated by stopping the study agent, they will be withdrawn.

12.2 Withdrawal Procedures: No further data will be collected for withdrawals. In the case of a partial withdrawal (e.g., treatment stopped due to adverse effects from the intervention), data collection will continue unless the participant indicates otherwise.

12.3 Termination Procedures: If the study is terminated, participants will be notified by telephone or mail. Any data collected during this pilot would be used after termination.

13.0 Risks to Participants

13.1 Foreseeable Risks: Possible immediate risks could be associated biometrics measures, blood draws, stool collections, acute gout flare during the study period, side effects from vitamin C, and risk of genetic testing.

- As our study collects biometrics such as weight, height, and waist circumference, as a measure of obesity, participants may experience emotional upset upon learning the results of their obesity measurement.
- Risks associated with blood draws could include bruising, nausea, or fainting.
- When collecting stool samples by participants, skin contamination with feces from the collection container could happen.
- The intervention, vitamin C may not be sufficient to lower SU and prevent acute gout flare.
- Serious adverse reactions of vitamin C includes nephrolithiasis and hemolysis. Vitamin C can increase production of oxalate resulting in the deposition of oxalate within the kidneys and the formation of oxalate stones within the renal tract. Acute renal failure because of calcium oxalate crystals has occurred in several patients receiving a single intravenous dose of ascorbic acid, ranging from 2.5 to 60 g. Patients with glucose-6-phosphate

dehydrogenase deficiency following ascorbic acid administration have been reported with hemolysis.

- Risks related to obtaining genetic information
- Transmission of SARS-CoV-2 virus

Following methods are in place to minimize risks mentioned above:

- At study visits, biometrics will be measured in a private room and study researchers will reassure study participants that all the information collected will remain private.
- When participants experience any signs of side effects from blood draw, blood draw will be discounted immediately. Researchers will place participants in a supine position and elevate the feet above the body. Basin will be prepared for possible emesis. Participants will be allowed to rest as long as necessary. Water, juice and/or a granola bar will be offered.
- Detailed instructions on stool collection will be provided in both English and Hmong, with verbal, written and silent video instructions. These explanations will include how to collect stool to minimize potential contaminations and avoid ingestion of collection liquid.
- Given a relatively short study period (8 weeks), medications for gout flare prophylaxis will not be provided routinely at the beginning of the study. Rather, at the beginning of the study, every participant will have articulated their preferred plan to respond to acute gout attack, should that happen. Study physicians could prescribe appropriate medications including colchicine 0.6 mg once to twice a day within 12-36 hours of gout flare onset, or oral nonsteroidal anti-inflammatory drugs (such as indomethacin 50 mg three times daily until the flare subsides or the pain is tolerable), or corticosteroids (prednisone 20 to 60 mg every day for 5 days) if patients do not have medications from their usual source of gout care.
- Participants will be assessed for eligibility prior to the enrollment visit and again at the visit, just before beginning the informed consent process. The exclusion criteria should minimize the occurrence of serious adverse reactions from vitamin C.
- We will explain the risks of genetic testing in the consent process and provide options for use and disclosure of genetic results in the consent form. We will explain that we will store their DNA in a locked laboratory until the end of the study and if they want, we can then destroy it. We will offer them additional options for future use of their de-identified DNA material, which they will indicate on their consent form, including their decision whether to agree or not agree with three options: 1) our research team or other research teams could analyze their un-identified DNA for any future research study; our research team or other research teams could analyze their un-identified DNA only for future research studies about genes

and medicines, without contacting them; and our contacting them about possible future studies. In addition, we will give them additional information about genetic research, including: that we are only analyzing for specific genes related to gout and gout medicines; we are not testing them for other diseases, such as tests for cancers, seizures, or psychiatric diseases; and that we will not report any incidental findings, including genetic relationships between people.

- To minimize transmission of SARS-CoV-2 virus, we will offer off-site study enrollment and data collection options. Methods include phone interview (using REDCap to interview participants) or a phone hybrid (participant fills out questionnaires on own using internet access and recruiter/interviewer would be on phone for assistance), and home collection of blood and saliva by research staff going to participants' homes with personal protective equipment (PPE).

13.2 Reproduction Risks: N/A.

13.3 Risks to Others: N/A.

14.0 Potential Benefits to Participants

14.1 Potential Benefits:

Participants may benefit from the study intervention (vitamin C) which may lower SU and gout symptoms. However, most participants (e.g., healthy adults) will not derive any direct benefits from participating in the study. Participants will learn about their laboratory results and other health measures, which may not provide them with significant benefit. In general, the research project has a societal benefit that can potentially improve the management of gout within the Hmong population as well as other populations.

Compensation of up to $150 USD will be provided for those who completed the study. This compensation is based on the amount of time spent in the study and will be pro-rated for partial completion of each phase at two levels- each associated with provision of a stool sample. Participants will be informed that they can discontinue participation in the study at any time and will be paid for the time they put in.

15.0 Statistical Considerations

15.1 Data Analysis Plan:

Genomic Analysis: We will collaborate with University of Minnesota Genomic Center (UMGC) to extract and purify genomic DNA, genotyping key variants on transporter genes which associated with urate absorption and reuptake, including *ABCG2* and *SLC22A12.*[16]

Microbiome Analysis: We will collaborate with UMGC to extract DNA from the stool sample to sequence whole-genome shotgun sequencing of the micrbiota. following the UMGC protocol.[26] Whole-genome shotgun sequencing data will be trimmed and processed for quality using SHI7[27] and strain-level taxa will be annotated by comparison to a public strain database. Microbiome features (alpha diversity, beta diversity, taxon relative abundance, gene functional category) will be analyzed, as described in a previous study.[28]

15.2 Power Analysis:

For an effect size of 0.5 mg/dL SU reduction from baseline by vitamin C[14], using a one-sided *t*-test, 80% power, type 1 error of 0.01, and 15% drop-out rate, 120 in adults with HU and/or gout (standard deviation, SD of 1.5 mg/dL[19]) and 60 adults without HU and gout (SD of 1 mg/dL[14]) are required.

15.3 Statistical Analysis:

Absolute SU changes from baseline to week 8 will be calculated using multiple linear regression adjusting for patients' characteristics including age, gender, BMI, renal function, and concomitant medications. Genetic variants and microbiome features will be tested with forward stepwise regression.

Overall differences in microbiome composition from baseline to week 8 will be compared using three principle axes of variation in the microbiome. A linear regression will be analyzed with a bacterial taxon as dependent and host factors as independent variables. The significance of association of a host factor will be determined by the test of the null hypothesis that the coefficient is equal to zero. For microbiome features, we will correct computed p-values for the total number of comparisons between dependent and independent variables using false discovery rate correction.

15.4 Data Integrity:

Data for this study will be entered into a REDCap database, which uses a MySQL database via a secure web interface with data checks used during data entry to ensure data quality. REDCap includes a complete suite of features to support HIPAA compliance, including a full audit trail, user-based privileges, and integration with the institutional LDAP server. The MySQL database and the web server will both be housed on secure servers operated by the University of Minnesota Academic Health Center's Information Systems group (AHC-IS). The servers are in a physically secure location on campus and are backed up regularly, with the backups stored in accordance with the AHC-IS retention schedule. Backup tapes are stored offsite. The AHC-IS servers provide a stable, secure, well-maintained, and high-capacity data storage environment, and both REDCap and MySQL are widely-used, powerful, reliable, well-supported systems. Access to the study's data in REDCap is restricted to the members of the study team by username and password.

16.0 Health Information and Privacy Compliance

16.1 Select which of the following is applicable to your research:

☐ My research does not require access to individual health information and therefore assert HIPAA does not apply.

☒ I am requesting that all research participants sign a HIPCO approved HIPAA Disclosure Authorization to participate in the research (either the standalone form or the combined consent and HIPAA Authorization).

☐ I am requesting the IRB to approve a Waiver or an alteration of research participant authorization to participate in the research.

Appropriate Use for Research:

☐ An external IRB (e.g. Advarra) is reviewing and we are requesting use of the authorization language embedded in the template consent form in lieu of the U of M stand-alone HIPAA Authorization. Note: External IRB must be serving as the privacy board for this option.

16.2 Identify the source of Private Health Information you will be using for your research (Check all that apply)

☐ I will use the Informatics Consulting Services (ICS) available through CTSI (also referred to as the University's Information Exchange (IE) or data shelter) to pull records for me

☒ I will collect information directly from research participants.

☐ I will use University services to access and retrieve records from the Bone Marrow Transplant (BMPT) database, also known as the HSCT (Hematopoietic Stem Cell Transplant) database.

☐ I will pull records directly from EPIC.

☐ I will retrieve record directly from axiUm / MiPACS

☐ I will receive data from the Center for Medicare/Medicaid Services

☐ I will receive a limited data set from another institution

☐ Other. Describe:

16.3 Explain how you will ensure that only records of patients who have agreed to have their information used for research will be reviewed.

N/A. We will not be accessing patients' health records.

16.4 Approximate number of records required for review: N/A.

16.5 Please describe how you will communicate with research participants during the course of this research. Check all applicable boxes

☐ This research involves record review only. There will be no communication with research participants.

☐ Communication with research participants will take place in the course of treatment, through MyChart, or other similar forms of communication used with patients receiving treatment.

☒ Communication with research participants will take place outside of treatment settings. For the safety and adherence to the vitamin C treatment, biweekly phone call contacts will be made.

16.6 Explain how the research team has legitimate access to patients/potential participants:

Several community researchers may have legitimate access to medical records by virtue of their occupation or practice site (physicians, pharmacists). This may be a likely mechanism by which subject recruitment may naturally occur through routine clinical interactions.

16.7 Location(s) of storage, sharing and analysis of research data, including any links to research data (check all that apply).

☐ In the data shelter of the Information Exchange (IE)

☐ Store ☐ Analyze ☐ Share

☐ In the Bone Marrow Transplant (BMT) database, also known as the HSCT (Hematopoietic Stem Cell Transplant) Database

☐ Store ☐ Analyze ☐ Share

☒ In REDCap (recap.ahc.umn.edu)

☒ Store ☐ Analyze ☒ Share

☐ In Qualtrics (qualtrics.umn.edu)

☐ Store ☐ Analyze ☐ Share

☐ In OnCore (oncore.umn.edu)

☐ Store ☐ Analyze ☐ Share

☒ In the University's Box Secure Storage (box.umn.edu)

☒ Store ☐ Analyze ☒ Share

☐ In an AHC-IS supported server. Provide folder path, location of server and IT Support Contact:

☐ Store ☐ Analyze ☐ Share

☐ In an AHC-IS supported desktop or laptop.

Provide UMN device numbers of all devices:

☐ Store ☐ Analyze ☐ Share

☐ Other. Describe:

Indicate if data will be collected, downloaded, accessed, shared or stored using a server, desktop, laptop, external drive or mobile device (including a tablet computer such as an iPad or a smartform (iPhone or Android devices) that you have not already identified in the preceding questions

☐I will use a server not previously listed to collect/download research data

☐I will use a desktop or laptop not previously listed

☐I will use an external hard drive or USB drive ("flash" or "thumb" drives) not previously listed

☐I will use a mobile device such as a tablet or smartphone not previously listed

16.8 Consultants. Vendors. Third Parties. De-identified data will be made publicly available during analysis and following publication as is the standard in peer-reviewed medical journals.

16.9 Links to identifiable data: There will be no links to identifiable data; they will be contained in a Box spreadsheet accessible only to minimal staff necessary to create the de-identified spreadsheet.

16.10 Sharing of Data with Research Team Members. Data will be shared on Box (box.umn.edu).

16.11 Storage and Disposal of Paper Documents: Paper documents will be stored in a locked cabinet in the PI's secure lab space and will be destroyed using a commercial confidential paper shredding business.

17.0 Confidentiality

17.1 Data Security: To ensure confidentiality, all people enrolled in the study will be assigned a study identification code. This code will be used on all study related data collection forms except for those on which the use of personal identifiers is mandatory (e.g., informed consent form). Forms that link the name of the participant and the study identification code will be kept in a locked cabinet inside a locked office under the control/supervision of PI and Co-PIs and in an electronic file stored on a password protected, strongly encrypted computer server that meets HIPAA guidelines. Access to study participants' identifiable

information will be limited to those that require this information such as the principal investigators or others who have direct contact with study participants, such as the bilingual/ bicultural research staff. Any personnel with data access will be identified to the University of Minnesota IRB and listed on the HIPAA form.

18.0 Provisions to Monitor the Data to Ensure the Safety of Participants

18.1 Data Integrity Monitoring.

Drs. Culhane-Pera, Lo, Straka, and Knights will meet at least once per month to discuss the progress of the study and to ensure that it is being conducted, recorded, and reported in accordance with the protocol, standard operating procedures, and applicable regulatory requirements. Specifically, these investigators will review the following items at these regular meetings:

- Protocol procedures described in section 5.2 are being followed appropriately.
- Data are being analyzed and stored according to the procedures and policies described in sections 16 and 17 of this document.
- Safeguards for vulnerable populations described in section 9.2 are being followed.

18.1.1 Duties of monitors: Drs. Culhane-Pera and Lo will be primary responsible for the patient's recruitment and data collection. Drs. Straka and Knights will be responsible for data storage, sample testing, data generation and analysis.

18.1.2 Extent and nature of monitoring: This study is NOT greater than minimal risk and does not require a DSMB or DSMC.

18.1.3 Monitoring procedures:

- Monitoring collection of samples and data: Drs. Culhane-Pera and Lo will work with community researchers to enroll subjects to determine whether procedures in section 9.2 are being followed and whether any unforeseen issues are arising during participant recruitment and sample and data collection. Drs. Straka and Knights will work with the study staff performing data entry and will ensure that they are double entering all data for assurance of concordance to avoid data entry errors.
- Monitoring the safety and adherence of the intervention: Drs. Culhane-Pera and Lo will work with community researchers to follow-up with participants as described in section 9.2 through phone call.
- Monitoring receiving and testing of samples: Drs. Straka and Knights will work with UMGC to ensure the samples are labeled with patient identifiers and processed correctly. The de-identified sample information will be stored in UMN Box.

18.1.4 Expected elements of the monitoring reports, the distribution plan, and expected follow-up:

- Expected elements of monitoring reports: The reports will contain a list of the monitoring procedures above and the outcomes of the performance of each procedure.
- Distribution plan: reports will be distributed to relevant research staff on a monthly basis.
- Expected follow-up: Any deviations from procedure requiring corrective action or adaptation of the study protocol will be communicated to the staff involved and will be reported to the IRB through a protocol amendment request as needed.

18.2 Database Protection.

Source documents including all paper and electronic records for all enrolled participants (i.e., informed consent forms, case report forms, laboratory reports, subject study binders, etc.) will be kept according to IRB policy. All paper records will be stored in a locked cabinet where the key is accessible to only the PI and designated personnel.

University of Minnesota’s REDCap system will be used to store all electronic records. REDCap is secured with password protection. The research staff will receive only coded information that is entered into the database under those identification numbers. Electronic communication with outside collaborators will involve only unidentifiable information. Access to the study's data in REDCap is restricted to the members of the study team by username and password.

18.3 Data Safety Monitoring.

- This study is NOT greater than minimal risk and does not require a DSMB or DSMC.
- On a regular basis, Drs. Culhane-Pera, Lo, Straka, and Knights will interview study staff working with clinicians to enroll subjects to determine whether procedures in section 9.2 are being followed and whether any unforeseen issues are arising during participant recruitment and sample and data collection and whether there is any indication that participants do not remain safe.
- As this is a minimal risk study, there is no specific safety data, or data regarding untoward events or efficacy that will be reviewed in a systematic way.
- All participants will be provided with the contact information of the investigators on the consent form for the study. Any harms or concerns reported by telephone calls from participants will be added to the monitoring reports described in section 18.1 and will be reported to the IRB for guidance.

- As this is a minimal risk study, the planned safety data to be collected consists of the monitoring reports above that will ensure that the procedures in sections 5.2, 9.2, 16, and 17 are being followed appropriately.
- Individual participants will be stopped from participating if they contact the investigative team and ask to have their samples removed from the study; although there is minimal risk of harm to participants foreseen in this study, if unforeseen complications arise or there are reports of harm to participants from study staff or directly from participants, the investigators will seek the guidance of the IRB on when and how best to stop the study.
- Who will review the data: This is a minimal risk study and does not require a DSMB or DSMC, so the investigators Drs. Culhane-Pera, Lo, Straka, and Knights will review the data.
- The frequency or periodicity of review of cumulative data: Monitoring reports will be generated and distributed on a monthly basis.
- Any conditions that trigger an immediate suspension of the research: there are no conditions contemplated that would lead to immediate suspension of the research, but if there is a report from study staff or directly from a participant that any aspect of the study is causing unexpected harm, the study will be immediately suspended pending guidance from the IRB.

19.0 Provisions to Protect the Privacy Interests of Participants

19.1 Protecting Privacy:

Potential participants are encouraged to bring up questions and concerns about study participation. Participants are informed that participation is voluntary; they can refuse to answer any questions or complete any study activities if they feel uncomfortable, and that they may discontinue participation in the study at any time.

To protect the privacy of individuals who choose to participate in this study, testing (questionnaires, biometrics, and biospecimen collection) will be completed in a private space, away from other people. Only one researcher will be with the participant at the time of testing for privacy.

All enrollees will receive a unique study ID code. The study identification (ID) code is how all collected data will be recorded. Forms linking codes to personal identification information for all participants will be secured. All the data will be kept safe and under locked access during and after participants have completed the study based on the database protection policy described below. With study participants' permission for future contact, identifiers will only be available to the PI, the co-investigators, and select key personnel, such as a study coordinator. However, for participants who do not wish to be contacted for future studies, their data will be de-identified and identifiers will no longer be available to co-investigators.

19.2 Access to Participants*:*

For participant safety and only with their permission, relevant medical records will be requested and reviewed for any adverse events reported by participants. Participants will be asked to sign a release authorization to grant access to these records. No other type of private records will be accessed for the study.

20.0 Compensation for Research-Related Injury

20.1 Compensation for Research-Related Injury: N/A. This study is minimal risk. Vitamin C is available as an over-the-counter nutraceutical supplement. Treatment plan for acute gout flare will be discussed during enrollment of the study. We will not provide medical treatments or cover the medical costs for acute gout treatment.

20.2 Contract Language: N/A.

21.0 Consent Process

21.1 Consent Process (when consent will be obtained):

Verbal consent will be obtained prior to any study procedures. The consent process and subsequent study procedures will take place at enrollment locations (describe in **Section 5 Procedures Involved** and **Section 22 Setting**). Interested Hmong community members with and without gout will be contacted by study staff (bilingual Hmong community researchers or staff researchers) by phone or email; study staff will explain the study, determine inclusion/exclusion criteria, and then schedule a time for consent and enrollment at Visit #1.

At Visit #1, community researchers will provide participants with a copy of the consent form via paper by mail or electronically through REDCap. Researchers will review all parts of the consent form, will answer any questions participants may have regarding participation in the study and will ask questions to ensure participants understand the consent information. Participants will be given the opportunity to privately consider participation if desired. If the participant agrees to join the study, he/she/they will provide verbal consent and researchers will record their response in REDCap.

There is time (waiting period) between recruiting/informing the prospective participants about the study and obtaining the verbal consent. There will be no built-in time (waiting period) between consenting and enrolling, unless people want to wait and consider their options before signing and starting the study. Only people who are interested in the study will be scheduled to attend the first study visit.

21.2 Waiver or Alteration of Consent Process (when consent will not be obtained): N/A.

21.3 Waiver of Written/Signed Documentation of Consent (when written/signed consent will not be obtained):

The intervention of the study is vitamin C at the typical over-the-counter dose. The biological samples (blood in an amount of no more than 3 ml per kg in an eight-week period, saliva, urine, and stool) collected in the study are no more than minimal risk. Since only adults will be enrolled in the study, no newborn dried blood spots will be collected. Paper or electronic HIPAA form and consent form will be provided to all participants according to the procedure described in the consent process.

21.4 Non-English-Speaking Participants:

This study recruits Hmong participants. Bilingual (English and Hmong) community researchers will recruit, consent, and collect necessary data from the participants who only speak Hmong. For participants who can speak English fluently, community researchers or research staff who are not Hmong may recruit, consent, and collect data from them.

Both English and Hmong consent form and HIPAA form will be available to the study participants. Hmong Informed consent will be translated by bilingual community researchers who have the Certification of Translation Accuracy certified by SoLaHmo Partnership for Health & Wellness, Community University Health Care Center.

21.5 Participants Who Are Not Yet Adults (infants, children, teenagers under 18 years of age): N/A.

Participants will be required to show a driver’s license or passport to verify age of 18 years or older. Participants who are under the age of 18 years will not be enrolled in the study.

21.6 Cognitively Impaired Adults, or adults with fluctuating or diminished capacity to consent: N/A.

21.7 Adults Unable to Consent: N/A.

22.0 Setting

22.1 Research Sites: All study visits (enrollment and final visit) will occur at specific locations, which include CUHCC, MCC, and possible Hmong community organizations (such as Hmong markets). Given concerns about COVID-19 transmission, we also offered the option of no in-person contact at participants’ homes.

22.2 Meeting at people’s homes is a possibility, in which case research team members and interested participants will wear personal-protective equipment, including masks, shields, gloves, and gowns. Other study activities including self-stool sample collections and phone calls will occur in participants’ homes.

NOTE: In response to reducing risk for COVID-19 infection for study participants, all the activities conducted at A locations (the participating clinic sites) will also be offered as an option for no in-person contact at participants' home (B locations). Methods include phone/online interview (using REDCap to interview participants) or a phone/online hybrid (participant fills out questionnaires on their own using internet access or paper copies and recruiter/interviewer would be on phone for assistance). The collection of blood, saliva, urine, and stool will be performed by participants at their home. Once the samples have been collected, participants will contact research staff and research staff will go to participants' homes with personal protective equipment (PPE) including masks, shields, gloves, and gowns to collect the samples. During the collection process, support from research staff will be provided if needed through phone or Zoom per participants' preference.

22.3 International Research: N/A

23.0 Multi-Site Research

N/A

24.0 Coordinating Center Research

The University of Minnesota is serving only as the Coordinating Center for this research study.

24.1 Role: The University of Minnesota will store the biological samples and study related information. Saliva and stool samples will be processed at University of Minnesota Genomics Center, and for the subsequent testing and analysis of the samples.

24.2 Responsibilities: All the study components will be collected at CUHCC or MCC. No actual study will be conducted at the UMN.

24.3 Oversight: N/A

24.4 Collection and Management of Data:
Consent forms will be processed by a study coordinator. Participant's name, MRN or patient ID, date of birth as recorded on the consent form will be retained for record-keeping. The participant's name, date of birth, MRN or patient ID, and test kit barcodes will be added to a spreadsheet maintained on box.umn.edu and accessible only to the PI and minimal staff necessary to create the de-identified database. Study records will be collected and stored using REDCap.

25.0 Resources Available

25.1 Resources Available:

- Study supervision and oversight: The study will be conducted by a team of researchers including faculty, students, trained community researchers, and study coordinators. Drs. Knights and Straka will oversee sample and data acquisition and delivery of samples to the UMGC; Drs. Culhane-Pera and Lo

are study physicians who will oversee the safety of participants and provide study-related counsel and advice to the PI and study coordinators.

- The community researchers working on this project are experienced and have worked on similar projects and with this unique population in the past. Established work practices are in place, staff meets regularly to discuss on-going studies, and supervisors are readily accessible for consultation on any unusual circumstances.
- Facilities: CUHCC is a federally qualified health center and also a department within the University of Minnesota's Academic Health Center. The clinic offers various health care programs. The patient populations include Hmong which can make the recruitment process easier. MCC is a federally qualified health center in St Paul, MN, where Drs Culhane-Pera and Lo work.
- Facilities: The UMGC will perform all testing of samples. The University of Minnesota Genomics Center is fully equipped to do any testing required on these samples. The UMGC provides genomics research services committed to advancing genomics in Minnesota. The UMGC maintains and acquires state-of-the-art instrumentation and offers an array of services including sequencing, expression, genotyping, nucleic acid extraction and related support. The UMGC strives to keep pace with the ever-broadening world of “omics” technologies (genomics, epigenomics, metabolomics), and to expand its role at the University and the wider biotech community. The UMGC is committed to providing the following support to researchers at the University of Minnesota and to the broader research community: (1) Providing genomics research services for UMN investigators and external companies. Although the primary goal of the UMGC is to provide a diverse portfolio of research services to UMN investigators, the UMGC also has a mandate to translate discoveries in genomics to the founding of knowledge-based companies in the State of Minnesota; (2) Assess and acquire emerging technologies to drive the expansion of biomedical and agricultural research. The efficient and well-planned evaluation and acquisition of new instrumentation is paramount in maintaining a competitive advantage for UMN researchers and external clients. (3) Advise clients on choosing appropriate technology for research goals.

26.0 References

1 Kuo, C. F., Grainge, M. J., Zhang, W. & Doherty, M. Global epidemiology of gout: prevalence, incidence and risk factors. *Nat Rev Rheumatol* **11**, 649-662, doi:10.1038/nrrheum.2015.91 (2015).

2 Zhu, Y., Pandya, B. J. & Choi, H. K. Prevalence of gout and hyperuricemia in the US general population: the National Health and Nutrition Examination Survey 2007-2008. *Arthritis and rheumatism* **63**, 3136-3141, doi:10.1002/art.30520 (2011).

3 Shreiner, A. B., Kao, J. Y. & Young, V. B. The gut microbiome in health and in disease. *Curr Opin Gastroenterol* **31**, 69-75, doi:10.1097/MOG.0000000000000139 (2015).

4 Cho, I. & Blaser, M. J. The human microbiome: at the interface of health and disease. *Nat Rev Genet* **13**, 260-270, doi:10.1038/nrg3182 (2012).

5 Guo, Z. *et al.* Intestinal Microbiota Distinguish Gout Patients from Healthy Humans. *Sci Rep* **6**, 20602, doi:10.1038/srep20602 (2016).

6 Zeevi, D. *et al.* Personalized Nutrition by Prediction of Glycemic Responses. *Cell* **163**, 1079-1094, doi:10.1016/j.cell.2015.11.001 (2015).

7 Wahedduddin, S., Singh, J. A., Culhane-Pera, K. A. & Gertner, E. Gout in the Hmong in the United States. *J Clin Rheumatol* **16**, 262-266, doi:10.1097/RHU.0b013e3181eeb487 (2010).

8 Portis, A. J. *et al.* High prevalence of gouty arthritis among the Hmong population in Minnesota. *Arthritis care & research* **62**, 1386-1391, doi:10.1002/acr.20232 (2010).

9 Yanyan Zhu, P., Bhavik J. Pandya, P. & Hyon K. Choi, M., DrPH. Comorbidities of Gout and Hyperuricemia in the US General Population: NHANES 2007-2008. *The American journal of medicine* **125**, 679-687 (2012).

10 Richette, P., Clerson, P., Perissin, L., Flipo, R. M. & Bardin, T. Revisiting comorbidities in gout: a cluster analysis. *Annals of the rheumatic diseases* **74**, 142-147, doi:10.1136/annrheumdis-2013-203779 (2015).

11 Richette, P. *et al.* 2016 updated EULAR evidence-based recommendations for the management of gout. *Annals of the rheumatic diseases* **76**, 29-42, doi:10.1136/annrheumdis-2016-209707 (2017).

12 Khanna, D. *et al.* 2012 American College of Rheumatology guidelines for management of gout. Part 2: therapy and antiinflammatory prophylaxis of acute gouty arthritis. *Arthritis care & research* **64**, 1447-1461, doi:10.1002/acr.21773 (2012).

13 FitzGerald, J. D. *et al.* 2020 American College of Rheumatology Guideline for the Management of Gout. *Arthritis & Rheumatology* **n/a**, doi:10.1002/art.41247 (2020).

14 Juraschek, S. P., Miller, E. R., 3rd & Gelber, A. C. Effect of oral vitamin C supplementation on serum uric acid: a meta-analysis of randomized controlled trials. *Arthritis care & research* **63**, 1295-1306, doi:10.1002/acr.20519 (2011).

15 Stamp, L. K. *et al.* Clinically insignificant effect of supplemental vitamin C on serum urate in patients with gout: a pilot randomized controlled trial. *Arthritis and rheumatism* **65**, 1636-1642, doi:10.1002/art.37925 (2013).

16 Wen, C. C. *et al.* Genome-wide association study identifies ABCG2 (BCRP) as an allopurinol transporter and a determinant of drug response. *Clin Pharmacol Ther* **97**, 518-525, doi:10.1002/cpt.89 (2015).

17 Roberts, R. L. *et al.* ABCG2 loss-of-function polymorphism predicts poor response to allopurinol in patients with gout. *Pharmacogenomics J* **17**, 201-203, doi:10.1038/tpj.2015.101 (2017).
18 Wallace, M. C. *et al.* Association between ABCG2 rs2231142 and poor response to allopurinol: replication and meta-analysis. *Rheumatology* **57**, 656-660, doi:10.1093/rheumatology/kex467 (2018).
19 Roman, Y. M. *et al.* The Impact of rs505802 for *SLC22A12* on Oxipurinol and Uric Acid Disposition in Hmong Patients on Allopurinol from the Genetics of Hyperuricemia Therapy in Hmong (GOUT-H) Study. *Clinical Pharmacology & Therapeutics* **101**, S5-S99, doi:10.1002/cpt.570 (2017).
20 Fonseca, W. *et al.* Uric acid pathway activation during respiratory virus infection promotes Th2 immune response via innate cytokine production and ILC2 accumulation. *Mucosal Immunol* **13**, 691-701, doi:10.1038/s41385-020-0264-z (2020).
21 Merad, M. & Martin, J. C. Author Correction: Pathological inflammation in patients with COVID-19: a key role for monocytes and macrophages. *Nat Rev Immunol* **20**, 448, doi:10.1038/s41577-020-0353-y (2020).
22 Merad, M. & Martin, J. C. Pathological inflammation in patients with COVID-19: a key role for monocytes and macrophages. *Nat Rev Immunol* **20**, 355-362, doi:10.1038/s41577-020-0331-4 (2020).
23 Hirsch, J. D. *et al.* Evaluation of an instrument assessing influence of Gout on health-related quality of life. *J Rheumatol* **35**, 2406-2414 (2008).
24 Major, T. J., Dalbeth, N., Stahl, E. A. & Merriman, T. R. An update on the genetics of hyperuricaemia and gout. *Nat Rev Rheumatol*, doi:10.1038/s41584-018-0004-x (2018).
25 FitzGerald JD, *et al*. 2020 American College of Rheumatology Guideline for the Management of Gout. *Arthritis Care Res (Hoboken)*. 2020 Jun;72(6):744-760. doi: 10.1002/acr.24180. Epub 2020 May 11. Erratum in: Arthritis Care Res (Hoboken). 2020 Aug;72(8):1187. Erratum in: Arthritis Care Res (Hoboken). 2021 Mar;73(3):458. PMID: 32391934.
26 Gohl, D. M. *et al.* Systematic improvement of amplicon marker gene methods for increased accuracy in microbiome studies. *Nat Biotechnol* **34**, 942-949, doi:10.1038/nbt.3601 (2016).
27 Al-Ghalith, G. A., Hillmann, B., Ang, K., Shields-Cutler, R. & Knights, D. SHI7 Is a Self-Learning Pipeline for Multipurpose Short-Read DNA Quality Control. *mSystems* **3**, doi:10.1128/mSystems.00202-17 (2018).
28 Vangay, P. *et al.* US Immigration Westernizes the Human Gut Microbiome. *Cell* **175**, 962-972 e910, doi:10.1016/j.cell.2018.10.029 (2018).
