# Supplementary material for: Hmong microbiome ANd Gout, Obesity, Vitamin C (HMANGO-C): A phase II clinical study protocol
Source: PLoS One. 2023 Feb 1;18(2):e0279830. doi: 10.1371/journal.pone.0279830 (PMC9891498; doi:10.1371/journal.pone.0279830)
Supplement: S2 File — (PDF) [file pone.0279830.s003.pdf]

Consent Form

Title of Research Study: <u>H</u>mong <u>M</u>icrobiome <u>AN</u>d <u>G</u>out, <u>O</u>besity, Vitamin <u>C</u> (HMANGO-C) Study

Research Team Contact Information:

We are the people doing this study. If you have any questions about the research study, results, or other concerns, please contact us.

Investigator Name: **Robert J Straka**<br>Investigator Departmental Affiliation: College of Pharmacy<br>Phone Number: 612-624-5663<br>Email Address: strak001@umn.edu	Study Staff (if applicable): **Ya-Feng Wen**<br>Phone Number: 612-443-0511<br>Email Address: wenxx164@umn.edu

<u>University of Minnesota (UMN):</u>
Robert J Straka, PharmD, College of Pharmacy
Dan Knights, PhD, Computer Science of Engineering
Ya-Feng Wen, PharmD, College of Pharmacy
Boguang Sun, PharmD, College of Pharmacy

<u>SoLaHmo Partnership for Health and Wellness</u>
Yeng Moua, MS (612) 440-4170
Bai Vue, MEd (612) 440-4170
Toua Yang, MS (612) 440-4170
Kathleen A Culhane-Pera, MD, MA
Muaj C Lo, MD
Shannon L Pergament, MPH, MSW

Money:

This research is being supported by money from the **Minnesota Partnership for Biotechnology and Medical Genomics grant #18.08,** called **"Targeting the gut microbiome to prevent the increasing incidence of obesity in immigrant populations"**.

Researchers can also get money from other places that might affect their personal interests. For ethical reasons, we give you this information so you can know about these “financial interests”.

Dr. Dan Knights is a Senior Scientific Advisor to Diversigen, a *business that is analyzing germs in stool, which is now owned by* OraSure. These interests have been reviewed and managed by the University of Minnesota in accordance with its conflict-of-interest policies.

The other researchers say they have no conflict of interest that relates to this study.

Key Information About This Research Study

This form is to help you decide if you want to be a part of a research study about gout, obesity, the microbiome (germs in stool or poop) and vitamin C.

What is research?

Doctors and researchers want you to be healthy and safe. There are important differences between research and healthcare:

- The goal of research is to learn new things to help groups of people in the future. Researchers learn things by following the same plan with many people in a study to see how well that plan works. You may or may not personally be helped by being in a research study. But by being in a research study, you might help other people in the future.
- The goal of healthcare is to help you get better or to make your life better. Doctors and you can make changes to your healthcare as needed. This is different from research.

Why am I being asked to be in this research study?

We are asking you to be in this study because:

- Both of your parents are Hmong
- You are a Hmong adult (18 years old or older)
- You have gout or high uric acid, or you do not have gout or high uric acid
- You are not pregnant or breastfeeding
- You can read English or Hmong
- You do not have diseases in the gut, type 1 diabetes, kidney failure on dialysis, liver failure, or a genetic disease called glucose-6-phosphate dehydrogenase (G6PD) deficiency.

What should I know about a research study?

- Someone will explain this research study to you.
- Whether or not you choose to be in the study is up to you.
- You can choose not to be in the study.
- You can agree to be in the study and change your mind later.
- What you choose will not be held against you.
- You can ask all the questions you want before you decide.

Why is this research being done?

- We know that Hmong people have more gout and high uric acid than other Minnesotans. But we do not know why.
- We know that Hmong people with gout avoid different foods or drinks to prevent gout pain. But we do not know why.
- We know that many Hmong people do not want to take a prescription medicine every day to prevent gout pain.

  So, we wonder if taking vitamin C, which is found in food, could help. We want to learn if vitamin C could lower uric acid and prevent gout pain.

We are doing this study to find answers to four questions:

1. **Does Vitamin C improve clinical outcomes in people with gout and in people without gout?**
   Does vitamin C lower uric acid, reduce gout-related symptoms, and obesity?
2. **If Vitamin C does lower uric acid, does it work differently in different people?**
   Do germs in people's stools (poop) affect how vitamin C lowers uric acid in blood?
3. **Why do some people have gout or high uric acid while other people do not?**
   Do germs in people's stool affect the amount of uric acid in their blood?
4. **Why do people with gout have different "trigger foods" that can cause painful gout attacks?**
   Is there a relationship between germs in people's stool and the foods that cause gout attacks?

So, we want to find out if vitamin C can lower uric acid, gout-related symptoms, in people with gout or high uric acid which are problems commonly found in people with obesity and other metabolic diseases. We also want to know how people's germs in their stool are linked to gout or high uric acid.

What will I need to do to be in the study?

You will take Vitamin C twice a day for 8 weeks. Before you start and after you finish taking Vitamin C (called Visit #1 and Visit #2), you will spend about 2 hours answering questions, and giving blood, urine, spit, and stool samples. For these "visits", we could meet you someplace, or we could talk with you on the phone while you stay home. Also, we will call you by phone about 3 times during the study to see how you are doing.

Is there any way that being in this study could be bad for me?

This study has low risks to you. But, there are some risks with some of the steps:

- **Blood draw**. When blood is taken, sometimes people may faint, feel dizzy or off balance for a short time, or they get a bruise. If this happens, we will help you at the clinic. If you need medical care beyond what we can do, your insurance will have to cover that cost.
- **Stool (poop) kit**. We will show you how to put a small amount of stool into a tube and send it to us. The tube should be kept away from children. People should not drink the liquid that comes in the tube, although the small amount is not thought to be dangerous.
- **Vitamin C**. Vitamin C is a natural vitamin in citrus fruits (like oranges and lemons). The body needs Vitamin C. Taking vitamin C is usually not harmful to people. But there can be rare side effects, if people take too much vitamin C or for people who have kidneys that do not work well. The doses which can be harmful are usually over 8,000 mg a day which is 8 times higher than what we are using in this study). These rare side effects may include bleeding, red blood cell breakdown, higher blood sugar levels,and formation of kidney stones. During the study, we will call you by phone to make sure you do not have any symptoms that relate to these side effects. If any medical care needed is more than what we can do, your insurance will have to cover that cost.
- **Acute gout attack**. There is a chance that the vitamin C may not lower uric acid and prevent acute gout pain. We will help you make plans to manage pain if you have acute gout flare.
- **Your Privacy**. We will keep your information private and secure. However, there is a risk that someone could get access to the data we have stored about you. If the data has something serious

about your health, it could be misused. For example, it could be used to make it harder for you to get or keep a job or insurance. There are laws against this kind of misuse, but they may not give full protection. We believe the chance of this is very small, but we cannot promise it will not happen. Your privacy and the privacy of your data are very important to us and we will make every effort to protect them.

- **Internet Databases**. We will keep the study data on a secure internet database. We will not keep your name or contract information on that same database. But there is a risk that someone could trace the information in a scientific database back to you since your genetic information is unique to you. We think the chance that someone will know who you are is very small. But the risk may grow in the future if people find new ways to trace information.

Will being in this study help me in any way?

This study may or may not help you or your family. But what we learn in the study might help Hmong people with gout in the future.

- We will tell you what we learn from all the people in the study. This will include the results from all the people taking vitamin C. We will mail you a summary and invite you to hear about what we learn from the study as a group.
- We will also tell you the results of your tests, including blood pressure, weight, height, waist circumference, blood tests (uric acid and kidney function), genetic tests, poop (stool) tests, COVID-19 antibody tests and results of Vitamin C on uric acid. Note that the COVID-19 test can only tell you that you “at some point” may have been infected with the virus. It does not tell you if you are currently testing positively or not.
- Remember, this is research, not clinical care. The research findings are not for your doctors to use or to treat you but fulfill our promise to share findings of your involvement in this study.

What if I do not want to be in this study?

You do not have to be in this study if you do not want to. This study is “voluntary”. This means it is up to you to choose to be in the study or not. If you do not want to be in the study, it will not affect your relationship with the researchers, your clinic or your doctor.

More Facts About This Research Study

How many people will be in the study?

We hope that 180 Hmong adults will be in this research study from Minneapolis/St. Paul and surrounding areas.

What do I need to do if I am in this research?

If you choose to be in this study, you will meet with us 2 times, 8 weeks apart at a clinic or someplace else, or talk with us by phone and use the internet.

- At Visit #1, you will:
  - Answer questions about your age, health, diet, allergies, medicines, and medical history.
  - Measure or tell us your blood pressure, pulse, height, weight, and waist.
  - Have 1-2 teaspoons of blood drawn if in person or prick your finger and put 5 drops of blood into 2 tubes if at home. We will test for uric acid, kidney function (to see how well your kidney is working), swelling, and COVID-19 antibody (to see if you had COVID-19 in the past).
  - Pee in a cup, for gout and kidney function.
  - Spit in a tube to provide us with your DNA, which is genetic material.
  - Take home 2 stool (poop) kits. We will tell you how to put stool in the kit and send it back to us. We will see what kind of germs are in the stool, which might affect uric acid level.
  - Take home a packet of vitamin C. We will ask you to take 1 pill (500 mg) two times a day for 8 weeks. We think that vitamin C might decrease uric acid levels.
  - This visit will last up to 2 hours.

- At home (after Visit #1):
  - You will collect two stool (poop) samples and mail it back to us.
    If Visit #1 is at home, you will mail us the blood, spit, urine, and stool samples or we will pick them up from you.
  - When we get your samples, we will add $75 to your Greenphire ClinCard.
  - You will answer a few questions every week about what you have eaten or drunk.
  - You will take 1 Vitamin C 500 mg pill two times a day for 8 weeks. (We will provide you with the Vitamin C.)
  - We will call you every two weeks to ask about vitamin C, any gout symptoms, and any side effects from vitamin C and answer any questions you may have.

- Eight weeks later at Visit #2, you will:
  - Answer questions about any changes in your health, diet, allergies, medicines, and history.
  - Measure or tell us your blood pressure, pulse, height, weight, and waist measured.
  - Have 1-2 teaspoons of blood drawn or prick your finger and put 5 drops of blood into 2 tubes. We will again test for uric acid, kidney function, swelling and history of COVID-19.
  - Pee in a cup, for uric acid and kidney function.
  - Take home 2 stool (poop) kits for germs in the stool.
  - This visit will last about 30 minutes.

- At home after Visit #2:
  - You will collect two final stool (poop) samples.

If Visit #2 is at home, you will mail the blood, spit, urine, and stool samples or we will pick them up from you. We will tell you how to do this.

- When we get the samples, we will add another$75 to your Greenphire ClinCard.
- We will send you the results when we have them.

During the 8-week study, we will call you three times (at the end of weeks 1, 4, and 7) to see how you are doing.

What happens if I say "Yes", but I change my mind later?

If you are in this research study, and later want to leave, you should tell us. Your choice not to be in this study will not affect your right for medical care now or in the future. If you choose to leave the study, we will talk to you about the reasons for leaving the study in case there are any safety concerns and any follow-up care, if needed. If you decide to leave the study at any time during the study, no further information will be sought about you for this study, however the investigators may use the data collected to that point of notice of withdrawal.

Can you tell me to leave the study?

We might ask you to leave the study before you finish it. If this happens, we will tell you why.

Will it cost me anything to be in this research study?

Being in this research study will not cost you anything. However, we will not pay for any health care services that you would normally pay for or any transportation to/from the visits.

What happens to the information collected for the research, including my health information?

We will do everything we can to keep your personal information and your study information safe and private, so only people doing the study can see it. The people who will see your information are the people at the university who watch to make sure we do a good job and do not break laws that protect your safety and privacy. This includes people with the Institutional Review Board (IRB), and the Quality Assurance Program of the Human Research Protection Program (HRPP). However, we cannot promise complete safety and privacy, as sometimes people do illegal things, like hack computers.

We will keep your information safe and private. We will give you a study number and use that number instead of your name on all your information (questionnaires, blood, urine, stool, and saliva samples). We will keep a record that links your name with your number in a Secure Storage (Box) managed by the University of Minnesota, which only the research staff can see. We will keep any of your written material we have collected (like consent form) in a locked cabinet in the researcher's secure lab space. When the study is over, a private paper shredding business will destroy all your papers. We will store all your electronic documents in the University of Minnesota secure cloud space (called REDCap).

Additional sharing of your information for mandatory reporting

If we learn about any of the following, we must report this information:

- Abuse of children or adults

- Infections
- Other diseases by state or US law
- Excessive use of alcohol or controlled substances for non-medical reasons during pregnancy

How will my information be used in publications and presentations?

We plan to share the group results of this study with the Hmong community, doctors, and researchers through talks, papers, journals, and the internet. When we share what we learned, we will never show any information that identifies you (such as your name, birthdate, address, phone number, email, and medical records number) as someone who was in the study. However, if you have a unique or rare condition that is not shared by many others, it is possible that some people may be able to know who you are even without using information that identifies you.

What will be done with my data and specimens when this study is over?

When the study is over, we will keep all the results without your identifying information. This is called deidentified data. Identifying information means name, birthdate, address, phone number, and email address. However, if you tell us that we can contact you for further studies, we will keep your contact information separate from your study results . Otherwise, all deidentified data and specimens will be securely stored if a case arises where future analysis within the scope of our study may be helpful. You have two choices of how you will allow us to analyze your data at the end of this consent form.

Genetic Information

We will test for DNA (genes) that relate to gout and response to medications. We will not do tests for any other genes, including those connected with cancers, seizures, other diseases, or family relations.

A federal law, called the Genetic Information Nondiscrimination Act (GINA), generally makes it illegal for health insurance companies, group health plans, and most employers to discriminate against you based on your genetic information. This law generally will protect you in the following ways:

- Health insurance companies and group health plans may not ask for your genetic information that we get from this research.
- Health insurance companies and group health plans may not use your genetic information when making decisions regarding your eligibility or insurance payments.
- Employers with 15 or more employees may not use your genetic information that we get from this research when deciding to hire, promote, or fire you or when setting the terms of your employment.

Be aware that this federal law does not protect you against genetic discrimination by companies that sell life insurance, disability insurance, or long-term care insurance.

Who else receives results?

We will post group results (but not participant specific information) about this study on a website for studies at http://www.ClinicalTrials.gov, as required by U.S. Law, [Section 801 of the Food and Drug Administration Amendments Act (FDAAA 801) For more information see CT.Gov Guidance.] This Web site will not include your name or any other specific information about you. The Web site may include a

summary of the results of this research. You can search this Web site at any time.

Who will be with me when I learn about the study?

Only the researchers will meet you as you decide to join the study. There might be other people who are also thinking about joining the study.

Who else can I talk to about the study?

The University of Minnesota IRB within the Human Research Protections Program (HRPP) has read and approved this study. If you want to ask them questions or talk privately with them about your research experience, call the Research Participants' Advocate Line at 612-625-1650 (Toll Free: 1-888-224-8636). You can ask for an interpreter. Or you can go to z.umn.edu/participants. We encourage you to call if:

- Your questions, concerns, or complaints are not being answered by the research team.
- You cannot reach the research team.
- You want to talk to someone besides the research team.
- You have questions about your rights as a research participant.
- You want to get information or provide input about this research.

Will I have a chance to provide feedback after the study is over?

At the end of this study, the HRPP may ask you to complete a survey that asks about your experience in the research. You do not have to complete the survey if you do not want to. If you do choose to complete the survey, your name will not be on your answers, so your responses will be "anonymous".

If HRPP does not ask you to complete a survey, but you would like to share feedback, you can contact the study team or the HRPP. See the "Investigator Contact Information" at the top of this form for study team contact information and see the above section for the HRPP contact information.

What happens if I am injured while participating in this research?

If you get injured by being in the study, we can give you first aid, or advice and support to get you the help you need. If you need medical care, your insurance will have to cover the cost, in the usual way that your insurance works. If you think that you have been harmed by the study, let us know right away.

Will I be compensated for my participation?

If you agree to join the study, we will pay you up to $150 for your time and effort. This includes $75 for completing Visit #1 and $75 for completing Visit #2.

We will give you a prepaid debit card called Greenphire ClinCard. It works like a bank debit card. We will give you the card when you start the study. After you send us a stool sample, we will add money to the card. You may use this card at any store that accepts MasterCard or you can use a bank machine to remove cash. However, there may be fees drawn against the balance of the card for cash withdrawals (ATM use) and inactivity (no use for 6 months). We will give you the ClinCard Frequently Asked Questions information sheet that answers common questions about the debit card. You will also receive a cardholder agreement. Be sure to read all of this information for details about fees.

The debit card system is administered by an outside company Greenphire. We will give your name and address to Greenphire and MasterCard, they will use this information only as part of the payment system. Your information will not be used for any other purposes and will not be given or sold to any other company. Greenphire and MasterCard will not receive any information about your health status or your study information.

This $150 payment is considered taxable income. If you have gotten $600 or more in one year from being in the University of Minnesota research studies, UMN must tell the Internal Revenue Service (IRS) and must send you a FORM 1099 (Miscellaneous Income) for you to report on your taxes.

Use of Identifiable Health Information

We will do everything we can to keep your personal information private and safe. If you agree to join this study, you agree to tell us your personal information. Personal information includes information about your health, and information that can identify you. For example, personal health information may include your name, address, birthdate, email and phone number or social security number. However, we cannot promise complete safety and privacy, as sometimes people do illegal things, like hack computers. Please read the HIPAA Authorization form that we have provided and discussed.

CONSENT

Optional Elements:

The following research activities are optional, meaning that you do not have to agree to them in order to join the study. Please write your initials to show if you agree or do not agree with each of these options.

Yes<br>I agree	No,<br>I do not agree	
_________	___________	Researchers can text me when money is on the Greenphire Clincard.
_________	___________	Researchers can contact my doctor about the medications I am taking now.
_________	___________	Researchers can contact me in the future about joining other research studies by these researchers.
		Researchers can keep and use my stool samples and my genes (DNA) from the spit samples without my identifying information to:
_________	___________	a. study the effects of genes on medicines.
_________	___________	b. study the effects of genes on diseases.

Please sign if you agree to be in this study. We will give you a copy of this signed consent form.

____ ___________________
Signature of Participant Date

____
Printed Name of Participant

____ ___________________
Signature of Person Obtaining Consent Date

____
Printed Name of Person Obtaining Consent

WITNESS STATEMENT:

The participant was unable to read or sign this consent form because of the following reason:

☐ The participant is illiterate
☐ The participant is visually impaired
☐ The participant is physically unable to sign the consent form. Describe:_________________________
☐ Other *(please specify)*: ____

Consent Form

For the Consent of Non-English Speaking Participants when an Interpreter is Used:
As someone who understands both English and the language spoken by the subject, I represent that the English version of the consent form was presented orally to the subject in the subject's own language, and that the subject was given the opportunity to ask questions.

____ ___________________
Signature of Interpreter Date

____
Printed Name of Interpreter

OR: Statement from a Non-Interpreter:
As someone who understands both English and the language spoken by the subject, I represent that the English version of the consent form was presented orally to the subject in the subject's own language, and that the subject was given the opportunity to ask questions.

____ ___________________
Signature of Individual Date

____
Printed Name of Individual
