# Supplementary material for: Hmong microbiome ANd Gout, Obesity, Vitamin C (HMANGO-C): A phase II clinical study protocol
Source: PLoS One. 2023 Feb 1;18(2):e0279830. doi: 10.1371/journal.pone.0279830 (PMC9891498; doi:10.1371/journal.pone.0279830)
Supplement: S3 File — (PDF) [file pone.0279830.s004.pdf]

UNIVERSITY OF MINNESOTA

PERMISSION TO USE HEALTH INFORMATION FOR RESEARCH
HIPAA[1] AUTHORIZATION FORM

IRB Study Number: STUDY00010406
Study Title: Hmong Microbiome And Gout, Obesity, Vitamin C (HMANGO-C) Study
Principal Investigator Name: Robert J Straka
Principal Investigator Mailing Address: 7-115B Weaver Densford Hall, 308 Harvard Street SE, Minneapolis, Minnesota 55455 USA

A. What is the purpose of this form?

The purpose of this form is to give your permission to us to use and share your health information for the research study listed above, and if we need your medical records, to give your permission to health care providers who are treating you to share your medical records with us for research. Your information may then be used by the research team for the research described in the Consent Form, and may also be shared by the research team with others, including those who support the research, have oversight over the research, or sponsor the research, as explained below. This form also describes the type of health information that will be used for the research. If you decide to give your permission and to participate in the research, you must sign this form and the Consent Form. You should be aware that once your health information is shared with others as described in this form, it may not be protected by privacy laws, and might be shared with others beyond those described in this form or the Consent Form.

B. What health information will be made available?

Health information about you to be used and shared for the research includes those items checked by the research team below:

☐Your medical records, which may include records from hospital and clinic visits, emergency room visits, immunizations, medical history and physical exams, medications, images and imaging reports, progress notes, psychological tests, EEG/EKG/ECHO reports, lab and pathology reports, dental records and financial records. These records may be used and shared for as long as the research continues.

☒ Information collected as part of this research study, including research procedures, research visits, and any optional elements of the research you agree to, all as described in the Consent Form. This information might not be part of your medical record, and may include things like responses to surveys and questionnaires, and information collected during research visits described in the Consent Form.

---

1 HIPAA is the Health Insurance Portability and Accountability Act of 1996, a federal law related to privacy of health information.

C. What about more sensitive health information?

Some health information is so sensitive that it requires your specific permission. If the research study you are participating in requires any of this sensitive information, the boxes below will be marked and you will be asked to initial to permit the information to be made available to the research team to use and share as described in the Consent Form.

☐ My drug and alcohol abuse, diagnosis and treatment records. ____(initial)
☐ My HIV/AIDS testing records.____ (initial)
☐ My genetic testing records._____ (initial)
☐ My mental health diagnosis or treatment records._____ (initial)
☐ My sickle cell anemia records._____ (initial)

D. Who will access and use my health information?

If you agree to participate in this research, your health information will be shared with:

1. The research team conducting the research described in the Consent Form, including any collaborating or affiliated research institutions involved in conducting the research described in the Consent Form;

2. Others at the University of Minnesota and M Health/Fairview who provide support for the research or who oversee research (such as the Institutional Review Board or IRB which is the committee that provides ethical and regulatory oversight of research at the University, systems administrators and other technical and/or administrative support personnel, compliance and audit professionals, individuals involved in processing any compensation you may receive for your participation, and others);

3. The research sponsor(s), any affiliates, partners or agents of the sponsor(s) involved in the research, organizations funding the research, and any affiliates, partners or agents of the funding organization(s) involved in the research;

4. Organizations who provide accreditation and oversight for the research team, and others who are authorized by law to review the quality and safety of the research (such as U.S. government agencies like the Food and Drug Administration, the Office of Human Research Protections, the Office of Research Integrity, or government agencies in other countries); and

5. Organizations that process any payments that may be made to you for participating in this study and any other individuals or organizations identified in the Consent Form.

E. Am I required to sign this form?

No, you are not required to sign this form. However, if you do not sign this form, you will not be able to participate in this research study. Treatment available outside of the study, payment for

such treatment, enrollment in health insurance plans and eligibility for benefits will not be impacted by your decision about signing this form.

F. Will I be able to look at my records?

It is possible that the research team may not allow you to see the information collected for this research study. However, you may access any information placed in your medical records after the study is complete.

G. Optional research activity

The research study that you are participating might have optional research activities associated with it, meaning that you do not have to agree to these activities in order to participate in the research study. Please indicate your willingness to participate in these optional activities and authorize use of your information from these optional activities as described below by placing your initials next to each activity.

Yes, I agree	No, I disagree	
☐	☐	Researchers can send text me reminders using when money is on the Greenphire Clincard.
☐	☐	Researchers can contact my doctor about the medications I am taking now.
☐	☐	Researchers can contact me in the future about joining other research studies by these researchers.
		Researchers can keep and use my stool sample samples and genetic my genes (DNA) sample and its genomic data from the spit samples without my personal identifying information for future research studies about to:
☐	☐	A) study the effects of genes on medicines
☐	☐	B) study the effects of genes on medical conditions

H. Does my permission for making my health information available for use and sharing ever expire?

No, there is no expiration date.

I. May I cancel my permission for making my health information available for use and sharing?

Yes. You may cancel your permission at any time by writing to the researcher at the address at the top of this form. If you cancel your permission, you will no longer be in the research study. You may want to ask someone on the research team if canceling will affect any research related medical treatment. If you cancel your permission, any health information about you that was already used and shared may continue to be used for the research study and any optional elements to the study to which you agreed above.

J. Signature

If you agree to the use and release of your personal health information as described in this form, please print your name and sign below. You will be given a signed copy of this form**.**

____

Research Participant's Name (print)
(*required even if signed by parent/legal representative*)

____ __________________

Research Participant's Signature Date
(required even if form is read to participant because he/she cannot read the form)

Parent or Legally Authorized Representative

If you agree to the use and release of the Personal Health Information of the Research Participant named above, please print your name and sign below.

____

Parent or Legally Authorized Representative's Name (print)

____

Relationship to the Research Participant

____ __________________

Parent or Legally Authorized Representative's Signature Date

Witness/Translator

My signature below documents that the information in the HIPAA Authorization Form was accurately explained (or read) to, and apparently understood by, the participant, and that authorization was freely given by the participant.

____
Witness/Translator Name (print)

____ ____________________
Witness/Translator Signature Date

Instructions for Researchers: Do not make any changes to this form other than the following items:

The IRB **will not** be confirming the accuracy of the information you complete on this form. The researchers are responsible for accurately completing the HIPAA Research Authorization as follows:

1. Section B: Mark all sources of health information that will be released to the research team from M Health or other providers.

2. Section C:
   a. Check the box ***only*** for each specific type of information that will be collected for this study
   b. Records for drug and alcohol abuse, diagnosis and treatment are records related to admissions to treatment centers; records for mental health diagnosis or treatment are records related to admissions to mental health units
   c. Obtain the participant's initials ***only*** *for the specific types of information checked*

3. Section G:
   a. Check the boxes indicating if there are optional research activities or not
   b. Obtain the participant's initial *only if the study involves optional research activity*

4. Section J: Obtain the participant's name, signature, and date; *complete subsequent signature lines* ***if applicable***

5. Provide the participant with a signed copy of the form

Note: This form allows you to check the boxes electronically. You can make a 'master version' of this form for this study with all pertinent boxes checked.
