# Supplementary material for: Hmong microbiome ANd Gout, Obesity, Vitamin C (HMANGO-C): A phase II clinical study protocol
Source: PLoS One. 2023 Feb 1;18(2):e0279830. doi: 10.1371/journal.pone.0279830 (PMC9891498; doi:10.1371/journal.pone.0279830)
Supplement: S4 File — (PDF) [file pone.0279830.s005.pdf]

Title of Research Study: <u>H</u>mong <u>M</u>icrobiome <u>AN</u>d <u>G</u>out, <u>O</u>besity, Vitamin <u>C</u> (HMANGO-C) Study

Research Team Contact Information:

Pab pawg kawm tshawb fawb xov tooj thiab email.

Peb yog pab pawg kawm tshawb fawb. Yog muaj lus nug txog kev kawm tshawb fawb no, yam lus tshawb tau los, los yog tias muaj lwm yam kev txhawj xeeb, thov hu rau peb pawg tshawb fawb rau ntawm no:

Tus thawj tshawb fawb lub npe: **Robert J Straka**<br>Tus thawj tshawb fawb chav kawm ntawv : College of Pharmacy<br>Xov tooj: 612-624-5663<br>Sau ntawv Email rau: strak001@umn.edu	Tus neeg ua hauj lwm nrog thawj tshawb fawb (if applicable): **Ya-Feng Wen**<br>Xov Tooj: 612-443-0511<br>Sau ntawv Email rau: wenxx164@umn.edu

<u>University of Minnesota (UMN):</u>
Robert J Straka, PharmD, College of Pharmacy
Dan Knights, PhD, Computer Science of Engineering
Ya-Feng Wen, PharmD, College of Pharmacy
Boguang Sun, PharmD, College of Pharmacy

<u>SoLaHmo Partnership for Health and Wellness</u>
Yeng Moua, MS (612) 440-4170
Bai Vue, MEd (612) 440-4170
Toua Yang, MS (612) 440-4170
Kathleen A Culhane-Pera, MD, MA
Muaj C Lo, MD
Shannon L Pergament, MPH, MSW

Nyiaj txiag:

Minnesota Partnership for Biotechnology and Medical Genomics grant #18.08, hu ua "Targeting the gut microbiome to prevent the increasing incidence of obesity in immigrant populations" yog kob nyiaj-pab txoj kev kawm tshawb fawb ntawm no.

Cov neeg tshawb fawb nrhiav tau nyiaj los ntawm ntau qhov chaw. Li no, peb yuav tsum tau qhia rau nej sawvdaws paub qhov tseeb tias qhov nyiaj no los qhov twg los kom muaj kev ncaj ncees.

Dr. Dan Knights yog ib tug thawj qhuab qhia nyob rau hauv Diversigen, ib lub koos haum ntsuas cov kab mob (bacteria) no,-uas tus tswv ntawm Diversigen hu ua Orasure. Raws txoj kev cai tshawb fawb thiab ua haujlwm, lub tsev kawm ntawv qib siab University of Minnesota yog tus saib xyuas txoj kev kawm tshawb fawb thiab them nyiaj kom haum raws txoj cai.

Lwm cov neeg ua hauj lwm txog kev kawm tshawb fawb no, tsis muaj tej yam dab tsi yuav los cuam tshuam ntawm txoj kev kawm tshawb fawb thiab them nyiaj no.

Lus Tseeb Ntawm Kev Kawm Tshawb Fawb No

Tsab ntawv no yuav pab qhia saib koj puas yuav tso cai koom tes nrog peb qhov kev kawm tshawb fawb no txog mob ko taw vwm, txoj kev rog muaj mob, cov kab nyob rau plab nyhuv zom quav, thiab Vitamin C.

Kev kawm tshawb fawb nws yog dab tsi?

Cov kws kho mob thiab cov kev kawm tshawb fawb xav kom yus noj qab nyob zoo, tsis muaj mob muaj nkeeg thiab tsis muaj teeb meem. Kev kawm tshawb fawb thiab kev nrhiav kev pab ntawm kho mob yeej muaj qee yam txawv:

- Qhov hom phiaj ntawm txoj kev kawm tshawb fawb no yog yuav nrhiav tau yam khoom tshiab pab tau ib pab neeg yav tom ntej. Cov kev kawm tshawb fawb no yuav muaj txheej txheem tib yam rau sawvdaws tas txhua tus uas los koom kom paub tias txheej txheem no puas pab tau li cas. Tej thaum yuav muaj yam yuav pab tau yus los yog tej thaum tsis muaj thiab. Txawm li cas los, yog koj koom qhov kev kawm tshawb fawb no, nyaj koj twb pab tau tib neeg lawm.
- Qhov hom phiaj ntawm kev kho mob yog kom pab koj noj qab nyob zoo. Cov kws kho mob yeej pab tau koj hloov kom pab raws li yuav pab tau. Qhov no yeej txawv ntawm kev kawm tshawb fawb.

Vim li cas thiaj li nug kom kuv koom hauv kev kawm tshawb fawb no?

Peb nug kom koj koom kev kawm tshawb fawb no vim tias:

- Koj niam thiab koj txiv nkawd ob leeg yog caj ceg Hmoob
- Koj yog ib tug neeg Hmoob laus, (18 xyoo rov sauv)
- Koj mob ko taw vwm, los yog koj muaj ntshav mob ko taw vwm siab. Tsis tas li ntawd, koj yog ib tug neeg uas tsis muaj mob ko taw vwm, los yog tsis muaj ntshav mob ko taw vwm siab
- Tam sim no koj tsis muaj me nyuam nyob hauv plab, los yog koj tsis pub mis rau me nyuam noj
- Koj nyeem tau lus Aaskiv los lus Hmoob
- Koj tsis muaj kab mob rau hauv plab nyhuv, tsis mob ntshav qab zib hom 1, raum puas lim ntshav lawm, mob siab, los yog mob roj ntsha hu ua Glucose-6-phosphate dehydrogenase (G6PD).

Kuv yuav tsum paub dab tsi txog kev kawm tshawb fawb no?

- Yeej muaj neeg yuav piav txog kev kawm tshawb fawb no rau koj.
- Txawm koj xav koom tsis koom nrog txoj kev kawm tshawb fawb no los yeej nyob ntawm koj xwb.
- Koj txiav txim tias tsis koom tes nrog txoj kev kawm tshawb fawb no los yeej tau.
- Txawm koj koom tes nrog txoj kev kawm tshawb fawb no lawm los, yog koj pauv siab tsis xav ua lawm lwm hnub los tau.
- Txawm txiav txim siab li cas los tsis muaj teeb meem dab tsi.
- Yog koj muaj lus nug li cas, los yeej nug tau ua ntej koj txiav txim siab koom tes nrog txoj kev tshawb fawb no.

Vim li cas thiaj ua kev kawm tshawb fawb no?

- Ntawm peb pej xeem Minnesota no, peb paub tias tib neeg Hmoob mob ko taw vwm thiab muaj ntshav mob ko taw vwm siab tshaj lwm haiv neeg. Tiam sis tsis paub tias yog vim li cas thiaj muaj mob zoo li no.

- Peb paub tias tib neeg Hmoob uas mob ko taw vwm caiv tau yam khoom noj khoom haus kom tus mob ko taw vwm thiaj li tsis huam tuaj. Tiam sis peb tsis paub tias yog vim li cas.
- Peb paub tias coov tug tib neeg Hmoob uas mob ko taw vwm tsis xav noj tshuaj txhua hnub tiv thaiv kom txhob mob ko taw vwm.

  Yog li no, peb xav paub tias yog noj Vitamin C, uas ib txwm nyob rau hauv khoom noj saib puas pab tau zoo. Peb xav paub seb Vitamin C no puas yuav ua rau cov ntshav mob ko taw vwm puas nqig thiab puas tiv thaiv kom txhob mob ko taw vwm.

Peb muaj plaub lo lus nug peb xav teb hauv txoj kev kawm tshawb fawb no:

1. **Vitamin C puas yuav pab tau tib neeg mob ko taw vwm thiab tib neeg uas tsis mob ko taw vwm?**
   Vitamin C puas yuav ua tau ntshav mob ko taw vwm nqig, ua tau kom kab mob ko taw vwm tsis txhob huam tuaj, thiab ua kom yus txhob rog rog heev.
2. **Yog Vitamin C ua rau ntshav mob ko taw vwm nqis tau, puas pab tau lwm tus tib neeg li cas thiab?**
   Cov kab nyhuv zom quav puas yuav pab tau Vitamin C ua kom ntshav ko taw vwm nqig thiab?
3. **Vim li cas ib txhia neeg mob ko taw vwm los sismuaj ntshav mob ko taw vwm siab tiam sis ib txhia neeg tsis zoo li ntawd?**
   Cov kab nyhuv zom quav puas yuav pab ua kom ntshav mob ko taw vwm nqig li cas?
4. **Vim li cas muaj tej yam khoom noj khoom haus ua rau ib txhia neeg mob ko taw vwm txawv ntawm ib txhia neeg thiab?**
   Ntawm kab zom quav thiab khoom noj khoom haus puas yuav muaj kev sib raug zoo.

Peb xav nrhiav saib seb Vitamin C puas ua ntshav mob ko taw vwm nqig thiab ua tau kom tus mob ko taw vwm txhob huam tuaj. Feem ntau, cov neeg rog heev thiab muaj lwm yam mob loj rau hauv yus lub cev. Tsis tas li ntawd, peb xav paub cov kab zom quav puas li cas kom mob ko taw vwm los sis ua rau ntshav mob ko tawm vwm siab.

Puas yuav muaj dab tsi kuv yuav tau ua yog xav koom tes nrog qhov kev kawm tshawb fawb no?

Koj yuav tau noj Vitamin C ob zaug tauj ib hnub kom kav 8 lub lim tiam. Ua ntej yuav pib koj yuav siv ob teev los teb peb cov lus nug, tso ntshav, ntsuas qaub ncaug thiab khaws koj cov quav rau peb. TPeb tuaj pab tau koj los yog hu xov tooj pab koj ua cov no los tau. Peb yuav hu xov tooj tuaj rau koj 3 zaug ntawm 8 lub lim tiam no, (xws li lim tiam 1, lim tiam 4, thiab lim tiam 7) saib seb koj puas nyob zoo li cas.

Puas Yuav muaj teeb meem li cas rau kuv thiab?

Kev kawm tshawb fawb no tsis muaj teeb meem loj thiab. Tab sis tsam muaj teeb meem raws li cov txheej txheem no.

- **Tso ntshav**: Thaum tso ntshav, muaj tej thaum tib neeg kiv taub hau los tsaus muag me ntsis los yog yuav muaj doog ntshav ntawm qhov chaw tso ntshav. Yog ntsib tau tej teeb meem no, peb yuav pab koj tom chaw kuaj mob. Yog tshaj yam peb pab tau ces koj li insurance yuav tsum tau pab.

- **Raj quav**: Peb yuav qhia koj tso quav rau lub raj, thiab xa rov qab tuaj rau peb. Khaws cia kom zoo thiab muaj cia deb ntawm me nyuam. Tsis txhob pub leej twg haus cov dej hauv lub raj no. Txawm haus me me lawm los tsis muaj teeb meem loj thiab.
- **Vitamin C**: Vitamin C ib txwm nyob rau txiv qaub xws li txiv kab ntxwv. Tib neeg lub cev yuav tsum tau Vitamin C thiaj li khiav tau zoo. Noj Vitamin C ib txwm tsis muaj teebmeem, tiam sis tsawg zaug tau ua tsis zoo rau tib neeg yog noj ntau heev tsis raws cai los sis tus tib neeg mob raum, tsis ua haujlwm zoo. 8,000 mg yog qhov uas koj yuav tsum txhob noj ntau tshaj. Nws ntau tshaj 8 npaug ntawm qhov uas peb yuav kom koj no rau ib hnub. Tsawg tsawg zaug muaj yog koj noj phiv los tsis haum no yuav ua rau koj los ntshav, ntshav puas, ntshav siab, thiab mob raum muaj pob zeb. Lub sijhawm tshawb fawb no, peb yuav siv xov tooj hu koj thiaj li paub tias koj puas muaj teeb meem li cas. Yog koj muaj mob tshaj li peb pab tau, koj li ntawv kho mob (insurance) yuav tau siv los pab koj.
- **Ko taw vwm mob huam**: Muaj tej thaum cov Vitamin C yuav tsis ua rau ntshav ko taw vwm nqig thiab ua kom mob ko taw vwm txhob huam tuaj. Peb yuav pab li peb pab tau kom tus mob txhob huam lawm.
- Peb yuav khaws koj cov ntaub ntawv kom zoo. Txawm khaws zoo npaum li cas los, tsawg zaug yeej muaj tau lwm tus neeg los pom koj cov ntaub ntawv thiab. Yog tias muaj tej yam ntawv tseem ceeb qhia txog koj tej kev mob tas los, tsam muaj tej tug siv cov ntawv no los ua tsis zoo, xws li siv los ua nyuaj rau yus txoj haujlwm los sis ua nyuaj rau yus daim ntawv kho mob (insurance) puas tau. Muaj kev cai lij choj los pab kom neeg txhob ua phem thiab, tiam sis tsam tsis pab thoob. Peb xav tias cov teeb meem no yuav tsawg heev tiam sis lees tsis tau tias yuav tsis muaj tej teeb meem kiag li. Koj cov ntaub ntawv tseem ceeb heev rau peb sawv daws, peb yuav ua txhua yam kom tiv thaiv koj cov ntaub ntawv tseem ceeb no.
- Internet databases: Peb yuav ua txhua yam kom tiv thaiv koj cov ntaub ntawv nyob rau hauv internet xws li (healthchart, email, passwords.) Peb yuav tsis khaws koj lub npe thiab chaw nyob ua ke li, tiam sis yuav muaj tej zaum uas tib neeg tshawb tau koj cov ntawv piav qhia txog koj li roj ntsha kom qhia rov qab los rau koj. Peb kuj xav hais tias muaj tsawg tsawg zaus uas lwm tus neeg sab nrauv thiaj li paub koj thiab koj cov ntaub ntawv xwb tiam sis yav tom ntej no yuav tsis paub thiab vim tib neeg nrhiav tau ntau txoj kev ua dhau cai los nyiag ntaub ntawm.

Koom qhov kev kawm tshawb fawb no puas yuav pab tau kuv?

Kev kawm tshawb fawb no tej zaum yuav pab tau thiab is tau koj thiab koj tsev neeg, tiam sis yam peb yuav tshawb tau kuj yuav pab tau rau peb Hmoob rau lub neej yav pem suab.

- Peb yuav qhia txog qhov uas peb kawm tau ntawm cov neeg hauv qhov kev kawm tshawb fawb no. Peb mam li xa ntawv tuaj rau koj saib thiab caw koj tuaj nrog peb tham txog qhov peb kawm tau.
- Peb yuav qhia qhov peb kawm tau txog koj cov ntshav, qhov koj siab li cas, qhov koj hnyav li cas, ntsuas duav, caj ceg roj ntsha, kuaj quav, tshawb pom ntawm kab tiv thaiv Covid-19 (saib koj puas tau muaj Covid-19 lub sijhawm dhau los).
- Nco ntsoov, qhov ntawm no yog kev kawm tshawb fawb, tsis yog kev pab soj ntsuam nyob hauv tsev kho mob. Tej no tsis yog rau koj tus kws kho mob saib xyuas koj tus kheej tiam sis yog kev kawm tshawb fawb qhia koj txog tej yam uas peb kawm tau.

Yog kuv tsis xav nyob rau hauv qhov kev kawm tshawb fawm no ne?

Koj yeej tsis tas nyob rau hauv qhov kev kawm tshawb fawb no. Kev koom tes yog nyob ntawm yeem xwb. Ntawm no txhais tau hais tias nyob ntawm tus tib neeg xav nyob los tsis xav nyob. Yog koj txiav txim tsis xav koom tes rau qhov kev kawm tshawb fawb no, yeej tsis muaj kev tu siab los xwm txheej rau koj los ntawm cov kev kawm tshawb fawb, hauv chaw kho mob thiab koj tus kws kho mob.

Cov Lus Tseeb Ntxiv Rau Kev Kawm Tshawb Fawb No

Muaj pes tsawg leej koom nyob rau hauv kev kawm tshawb fawb no?

Peb xav nrhiav kom tau 180 tus tib neeg Hmoob uas muaj 18 xyoo rov sauv nyob hauv Minneapolis/St. Paul thiab ib puag ncig ob lub zos no.

Kuv yuav tau npaj dab tsis thiaj li koom tau hauv qhov kev kawm tshawb fawb ntawm no?

Yog kuv koom rau hauv qhov kev kawm tshawb fawb no, yuav tau npaj:

Yog koj xav koom hauv qhov kev kawm tshawb fawb no, koj yuav tau ntsib thiab nrog peb tham 2 zaug, 8 lub lim tiam sib nrug tom peb lub tsev kho mob los yog qhov chaw sib ntsib.

- Sib ntsib zaum #1:
  Sib ntsib zaum #1, koj yuav tsum:
  - Teb cov lus nug txog koj lub hnub yug, noj qab nyob zoo li cas, noj haus li cas, muaj khaub thuas li cas, noj tshuaj dabtsi, thiab keeb kwm muaj mob li cas.
  - Ntsuas koj cov ntshav siab li cas, lub plawv dhia li cas, koj siab li cas, koj hnyav li cas, thiab koj lub duav loj li cas.
  - Muab 5 teev ntshav tso rau ib lub raj kom tau ob lub raj ntshav. Cov ntshav no yuav siv los qhia txog ntshav mob ko taw vwm, qhia saib ob lub raum puas khiav zoo, saib seb koj lub nrog cev puas o, thiab ntsuas saib koj puas muaj roj ntsha tua kab mob COVID-19.
  - Tso zis hauv khob, peb yuav kuaj ntshav mob ko taw vwm thiab kuaj raum khiav li cas.
  - Nti qob ncaug hauv lub raj qov ncaug thiaj li ntsuas tau caj ceg roj ntsha.
  - Nqa ob lub raj mus ntim quav rau 2 hnub tom ntej. Peb yuav qhia cov txheej txheem txog ntim quav thiab xa rov qab rau peb li cas thiaj li xa dawb. Peb yuav tshawb kab zom quav saib seb puas pab los tsis pab koj cov ntshav mob ko taw vwm.
  - Peb yuav muab ib pob Vitamin C rau koj nqa mus tsev. Peb yuav qhia kom koj noj 1 lub tshuaj, ob zaug rau ib hnub kom txog 8 lub lim tiam.
  - Hnub muaj kev sib ntsib no yuav ntsib li 2 teev.

- Tom qab sib ntsib zaum #1 koj yuav tsum:
  Koj yuav khaws koj cov quav rau ob lub raj thiab xa koj cov ntshav, qaub ncaug, thiab zis tuaj rau peb los yog peb mam tuaj nqa.
  - Thaum peb tau koj cov quav, cov zis, quav ncaug, thiab cov raj ntshav lawm peb mam li ntxiv $75 rau koj daim Greenphire ClinCard.
  - Koj yuav teb lus nug txog zaub mov thiab dej haus rau txhua lub lim tiam.
  - Koj yuav noj ib ntsia Vitamin C, ob zaug rau ib hnub kom txog 8 lub lim tiam. Ib ntsia Vitamin C muaj 500 mg nyob rau hauv thiab peb mam li muab cov Vitamin C no rau koj.

- Ob lub lim tiam twg, peb yuav hu koj ib zaug los nug koj saib koj noj Vitamin C li cas, mob ko taw vwm li cas, thiab teb koj cov lus nug.

- 8 lub lim tiam tom qab sib ntsib zaum 2, koj yuav tsum:
  - Teb cov lus nug saib puas muaj dabtsis pauv ntawm koj txog qhov noj qab nyob zoo, zaub mov thiab dej haus, muaj khaub thuas li cas, noj tshuaj dabtsi, thiab keeb kwm muaj mob li cas.
  - Ntsuas koj cov ntshav siab li cas, koj lub plawv dhia li cas, koj siab li cas, koj hnyav li cas, thiab koj lub duav loj li cas.
  - Muab 5 teev ntshav tso rau ib lub raj kom tau ob lub raj ntshav. Cov ntshav no yuav siv los qhia txog ntshav mob ko taw vwm, qhia saib ob lub raum puas khiav zoo, saib seb koj lub nrog cev puas o, thiab ntsuas saib koj puas muaj ntshav tua kab mob COVID-19.
  - Tso zis rau hauv khob, peb yuav kuaj ntshav mob ko taw vwm thiab kuaj saib raum khiav li cas.
  - Nqa ob lub raj mus ntim quav.
  - Qhov sib ntsib sib tham no yuav ntev li 30 feeb.

- Tom qab sib ntsib zaum #2:
  Koj yuav khaws ob raj quav rau peb. Peb mam li qhia saib koj yuav ua li cas rau cov raj quav, cov raj ntshav, cov qob ncaug, thiab cov zis no thiaj li txog peb.
  - Thaum peb tau cov quav, cov ntshav, cov quab ncaug, thiab zis lawm, peb mam li tso $75 rau koj daim Greenphire ClinCard.
  - Peb mam li xa cov ntaub ntawv uas qhia txog qhov peb kawm tau los ntawm kev kawm tshawb fawb no rau koj.

Ntawm 8 lub lim tiam hauv qhov kev kawm tshawb fawb no, peb yuav hu koj 3 zaug rau (lim tiam 1, 4 thiab 7) kom paub seb koj zoo li cas.

Yog kuv txiav txim siab xav koom, tiam sis yog ho pauv siab tom qab ne?

Yog yus txiav txim siab koom, tab sis ho pauv siab lawm, thov hais qhia rau peb paub. Pauv siab li no tsis muaj teeb meem li, tsis muaj dab tsi yuav tshwm sim ntawm koj txoj kev nrhiav kev pab hauv tsev kho mob los yog ntawm koj tus kws kho mob. Yog tias koj xav tawm no, peb xav nug txog vim li cas thiab puas muaj kev nyuaj siab los sis ntxhov siab li cas thiab koj puas xav tau kev pab li cas. Yog tias koj xav tawm thaum twg, peb yuav nres qhov kev kawm tshawb fawb rau koj thaum ntawd, tiam sis peb cov kws tshawb fawb tseem yuav siv koj cov khoom kom txog thaum koj tawm lawm.

Puas muaj zaum puav tias yuav rho kuv tawm ntawm kev kawm tshawb fawb no?

Yuav muaj zaum puav yuav tau rho neeg tawm ntawm peb txoj kev kawm tshawb fawb no. Yog muaj li no, peb mam qhia rau koj yog vim li cas.

Kuv puas yuav tau them dab tsi yog nkag rau hauv kev kawm tshawb fawb no?

Nyob rau hauv kev kawm tshawb fawb no tsis yuav raug nqi li cas. Yog koj muaj mob lub sijhawm uas koj koom qhov kev kawm tshawb fawb no, peb yuav them tsis tau koj cov nqi. Peb yuav tsis them tsheb thauj koj tuaj ntsib peb.

Yuav ua li cas rau kuv cov ntaub ntawm hauv qhov kev kawm tshawb fawb thiab kuv cov ntaub ntawv kho mob?

Peb yuav khaws koj cov ntaub ntawv tseem ceeb kom zoo rau hauv peb cov kws tshawb fawb uas khiav haujlwm raws cai tom tsev kawm ntawv qij siab University of Minnesota. Koos haum luam ntawv thiab khaws koj cov ntaub ntawv xws li Institutional Review Board (IRB), cov thawj saib xyuas txog txoj cai lij choj ntawm kev kawm tshawb fawb thiab cov neeg saib xyuas kom peb ua txhua yam li peb paub (Quality Assurance Program of the Human Research Protection program (HRPP)). Txawm ua zoo npaum li no los peb lees tsis tau hais tias peb yuav ua tau li siab xav.

Peb yuav khaws koj cov ntaub ntawv kom zoo. Peb yuav koj lub npe thiab txhua yam khoom (ntaub ntawm, quav, ntshav, quab ncaug, thiab zis) qhia txog koj tus kheem ntawm koj cov ntaub ntawv. Peb yuav khaws txhua yam tseem ceeb xauv cia kom zoo, zoo li zoo tau. Tus yuav tsom kwm saib xyuas tej no yog peb pab kws tshawb fawb no thiab lub tsev Kawm Ntawv Qij Siab University of Minnesota. Peb yuav khaws tag nrho txhua yam ntaub ntawv xws li tsab ntawv no rau hauv ib lub qhov chaw zoo. Thaum tej haujlwm no tag, peb yuav nrhiav ib tug contractor muab tej ntaub ntawv no txiav pov tseg kom tag. Txhua yam ntaub ntawv tseem ceeb peb yuav khaws zoo rau hauv computer nyob rau hauv University of Minnesota cloud space (REDCap.)

Peb yuav cia lwm tus siv koj cov ntaub ntawv tseem ceeb no thiab

Yog tias peb paub txog yam no, peb yuav tsum qhia rau cov neeg tuav txoj cai:

- Kev tsim txom me nyuam thiab lwm tus neeg
- Kis kab mob
- Lwm yam kab mob raws li kws lij choj nyob rau xeev Minnesota thiab Teb Chaws America hais ntawd
- Kev haus dej haus cawv los sis noj tshuaj tsis raws kev pab thaum muaj plab me nyuam

Nej yuav siv kuv cov ntaub ntawv li cas thaum tshaj tawm txog qhov kev kawm tshawb fawb no?

Peb npaj nthuav qhia txog qhov peb kawm tau ntawm qhov kev kawm tshawb fawb no rau pej xeem Hmoob, kws kho mob, nrog lwm tus kws tshawb fawb tham, sau ntawv thiab siv internet los qhia txog tej no. Yog thaum peb qhia tawm txog qhov peb kawm tau lawm, peb yuav tsis tuav koj lub npe kom neeg paub koj (xws li koj lub npe, hnub yug, chaw nyob, xov tooj, email, lossis ntaub ntawv kho mob) qhia tau hais tias koj nyob rau hauv peb txoj kev kawm tshawb fawb no. Tiam sis, yog hais tias koj muaj ib tug mob txawv txawv ces tsam ib co tib neeg ho yuav paub tias yog koj thiab.

Thaum kev tshawb fawb no tag lawm, kuv cov ntaub ntawv yuav mus rau qhov twg xwb?

Thaum kev kawm tshawb fawb no tiav tag lawm, peb yuav khaws tas nrho koj cov ntaub ntawv cia, tsis tso koj lub npe, hnub yug, chaw nyob, xov tooj thiab email rau qhov twg li. Cov ntaub ntawv tej ub no siv hauv kev kawm tshawb fawb hu ua “deidentified data”. Peb yuav khaws koj li xov tooj thiab chaw nyob

rau lwm qhov chaw txawv ntawm koj cov ntaub ntawv kom tau nrog koj tham yav tom ntej. Tsis tas li ntawd, yog koj qhia rau peb hais tias koj xav kom peb tiv tauj koj txog kev kawm tshawb fawb yav tom ntej, peb yuav khaws koj cov ntaub ntawv rau ib qho chaw txawv kom tsis txhob sib xyaw yam qub ntawm yam tshiab. tej zaum peb yuav khaws los muab koj cov ntaub ntawv piav roj ntsha pov tseg. Peb yuav cia koj xaiv rau ib daig ntawv saib seb koj yuav tso cai li cas thaum yuav kos npe ntawm daim ntawv no. Yog tsis xav kom peb tiv tauj koj yav tom ntej txog kev kawm tshawb fawb, tas nrho ntaub ntawv uas koj ho tau muab rau peb, peb yuav muab pob tseg. Peb yuav xav kom koj tso cai li cas hauvv daim ntav no saib seb koj yuav txiav txim siab li cas.

Ntaub ntawv roj ntsha

Peb yuav ntsuas koj li roj ntsha txuas tau nrog kev mob ko taw vwm thiab kev sib txuam nrog tshuaj uas koj tau noj. Peb yuav tsis tshawb fawb txog lwm hom ntawm koj li roj ntsha suav nrog cancer, qaug dab peg, los sis lwm hom mob los sis caj ceg ntawm tsev neeg li keeb kwm.

Raws kws lij choj, hu ua Genetic Information Nondiscrimination Act (GINA), hais mas ev sas las, lwm lub koos haum kho mob, thiab yus txoj haujlwm cais tsis tau koj los ntawm koj li roj ntsha vim hais tias qhov ntawv txhaum cai lawm. Kws lij choj yuav tiv thaiv koj li no:

- (Ev sas las) kho mob thiab lwm lub koos haum kho mob yuav nug tsis tau cov ntaub ntawv tshawb fawb no li.

- Ev sas las kho mob thiab lwm lub koos haum siv tsis tau koj cov ntaub ntawv piav txog koj li roj ntsha los xaiv seb yuav txiav txim siab li cas.

- Yog koj lub koos haum ua haujlwm muaj tshaj li 15 tus tib neeg, lawv yuav siv tsis tau koj cov ntaub ntawv roj ntsha ntawm kev kawm tshawb fawb no los ntiav koj, txhawb nqa koj tej haujlwm, los sis ncaws koj tawm ntawm haujlwm.

Ceeb toom tias txoj cai tiv thaiv tsis tau koj yog hais tias life ev sas las, disability ev sas las thiab ev sas las nyob tsev laus xav siv koj cov ntaub ntawv roj ntshav.

Leej twg thiaj yuav paub txog tej yam uas peb kawm tau ntawm qhov kev tshawb fawb no?

Peb yuav qhia txog tej yam uas peb kawm tau ntawm qhov kev tshawb fawb no (tiam sis tsis tso npe thiab lwm yam uas kom neeg taug qab tau txog koj) txog kev kawm tshawb fawb no nyob rau hauv website qhia txog kev kawm tshawb fawb http://www.ClinicalTrials.gov, raws kev cais kws lij choj, [Section 801 of the Food and Drug Administration Amendments Act (FDAAA 801) Yog xav paub ntxiv, mus rau CT.Gov Guidance.] Lub website no yuav tsis nthuav tawm lus txog koj tus kheej thiab koj li ntaub ntawv kom neeg taug qab txog koj. Lub website no yuav qhia me ntsis txog kev kawm tshawb fawb no. Xav nkag tshawb lub website no thaum twg lo yeej tau.

Leej twg puas yuav nrog kuv thaum kuv xyaum txog kev kawm tshawb fawb no?

Pab kws tshawb fawb yuav nyob nrog koj pab qhia koj txog txhua yam ntawm kev kawm tshawb fawb no. Yog muaj tej tug es xav koom nrog peb nyob hauv kev kawm tshawb fawb no ces lawv yuav nyob hauv chav nrog peb sawvdaws thiab.

Puas muaj lwm tus tib neeg uas kuv tham nrog tau txog kev kawm tshawb fawb no thiab?

The University of Minnesota IRB within the Human Research Protections Program (HRPP) has read and approved this study. If you want to ask them questions or talk privately with them about your research experience, call the Research Participants' Advocate Line at 612-625-1650 (Toll Free: 1-888-224-8636). You can ask for an interpreter. Or you can go to z.umn.edu/participants. We encourage you to call if:
Koos Haum IRB nyob rau hauv Tsev Kawm Ntawv University of Minnesota muaj kev kawm pab cuam txaus siab txais tos peb kev kawm tshawb fawb no lawm. Yog tias koj muaj lus nug li cas xav nrog lawv tham, koj ib leeg xwb, hu rau Research Participants' Advocate Line at 612-625-1650 (Hu dawb: 1-888-224-8636). Koj tiv tauj ib tug txhais lus los tau. Los sis koj mus rau lub website no los tau thiab z.umn.edu/participants. Peb xav kom koj hu lawv yog tias:

- Koj cov lus nug, kev txhawj xeeb los sis muaj kev yws los sis peb teb tsis tau koj cov lus nug.
- Yog koj hu tsis tau peb.
- Koj xav nrog lwm tus tib neeg txawv ntawm peb pawg tshawb fawb no tham.
- Koj muaj lus nug txog koj txoj cai ntawm kev kawm tshawb fawb no.
- Koj xav paub txog kev kawm tshawb fawb no los sis koj muaj qee yam zoo tshaj no yuav qhia rau peb

Nej puas yuav kam kuv qhia kuv lub tswv yim rau nej thaum uas peb kawm qhov no tag lawm.

Tom qab ntawm txoj kev kawm tshawb fawb no, HRPP yuav nug koj saib seb puas kam muaj koj li kev soj ntsuam nug txog kev paub ntawm kev kawm tshawb fawb no. Nyob ntawm koj saib seb koj puas kam teb ib qho lus nug. Yog tias koj xav tso koj cov lus no, peb yuav tsis tso koj lub npe ntawm daim ntawv thiab tsis muaj leej twg yuav paub.

Yog tias HRPP tsis muaj daim ntawv soj ntsuam rau koj, tiam sis koj kam tso lus thiab koj li tswv yim rau peb paub, koj hu tau rau HRPP. Ntawm nqe #1 thawj lus muaj "Investigator Contact Information" muaj xov tooj thiab email.

Yog kuv raug mob lub sijhawm koom tes nrog qhov kev kawm tshawb fawb no ne?

Yog koj lam raug mob lub sij hawm no, peb yuav cawm koj thiab pab koj los sis txhawb koj los ntawm koj txoj kev pab. Yog koj xav mus kho mob, koj lub ev sas las yuav tsum pab them ib yam li ev sas las ib txwm tau pab koj. Yog koj xav tias koj raug mob los ntawm qhov kev kawm tshawb fawb no, qhia peb paub sai li sai tau.

Lawv puas yuav them kuv yog kuv koom qhov kev tshawb fawb no?

Yog koj txiav txim siab koom qhov kev kawm tshawb fawm no, peb yuav them txog $150 rau koj lub sij hawm thiab koj txoj kev txaus siab zaum no. Koj yuav tau txais $75 thaum koj ntsib peb zaum #1. Koj yuav tau txais $75 ntxiv thaum koj ntsib peb zaum #2.

Peb yuav muab daim card Greenphire Clincard rau koj. Siv tau tib yam li koj daim bank debit card. Thiab peb yuav muab daim card rau koj ua ntej thaum pib qhov kev kawm tshawb fawb. Thaum koj xa koj raj quav thawj zaug rau peb, peb yuav tso nyiaj rau hauv koj daim card. Koj siv tau daim card no ntawm

qhov chaws uas txais Mastercard los sis yog koj xav rho nyiaj siv ces, daim no siv tau tom ATM thiab. Yog rho nyiaj ces tej thaum lawv yuav txiav me ntsis tawm. Yog tias koj tsis siv daim card no ntev tshaj 6 lub hli ces lawv yuav txiav tawm me ntsis thiab. Peb mam li muab daim ntawv qhuab qhia txog daim card no rau koj. Nco ntsoov nyeem tag nrho cov lus qhia txog txiav nyiaj tawm ntawm daim card no. Daim card no muaj tswv saib xyuas hu ua Greenphire. Peb yuav muab koj lub npe thiab chaw nyob rau lawv thiab rau Mastercard. Lawv mam li siv cov ntaub ntawv no them koj. Lawv tsis pub koj cov ntaub ntawv rau leej twg li tsuas yog them koj xwb. Peb yuav tsis pub leej twg paub txog koj cov ntaub ntawv uas koj muab rau peb kev tshawb fawb zaum no.

Qhov $150 no yeej yuav tau them se (tax) vim yog haujlwm lawm. Yog tias koj tau tshaj $600 los ntawm University of Minnesota rau ib lub xyoo, lawv yuav qhia Internal Revenue Service (IRS). Yog tshaj $600, lawv yuav xa ib daim ntawv 1099 (Miscellaneous Income) rau koj ua se.

Koj cov ntaub ntawv kho mob

Peb yuav khaws koj cov ntaub ntawv kom zoo, tsis pub leej twg sab nrauv paub thiab siv ua phem. Yog koj koom qhov kev tshawb fawb no, koj tso cai rau peb paub txog koj tus kheej, xws li, koj cov ntaub ntawv kho mob uas muaj koj lub npe, chaw nyob, hnub yug, email, xov tooj, thiab social security number. Txawm peb ua zoo npaum li no los tej lub sij hawm tseem muaj neeg phem nyiag tau thiab. Tab sis, peb yuav tsum ceev cov ntaub ntawv no zoo li zoo tau.

Tso Cai

Koj xav teb cov lus nug no thiab tsis xav teb los tau

Cov lus nug no yog rau koj teb thiab tsis teb los nyob ntawm koj. Qhov nov tsis cuam tshuam txog qhov koj los koom qhov kev kawm tshawb fawb no. Thov kos koj lub npe hauv qab no qhov Tso Cai, los qhov Tsis Tso Cai.

Tso Cai   Tsis Tso Cai

_________ ___________ Kws tshawb fawb ntaus ntawv hauv xov tooj tuaj rau kuv txog nyiaj hauv koj daim Greenphire Clincard.

_________ ___________ Kws tshawb fawb nrog kuv tus kws kho mob tham txog kuv cov tshuaj uas kuv noj lub sijhawm no.

_________ ___________ Kws tshawb fawb yuav hu rau kuv txog lwm qhov kev kawm tshawb fawb yav tom ntej.

Kws tshawb fawb siv tau kuv cov quav thiab caj ceg roj ntshav tom qab qhov kev kawm tshawj fawb no xaus. Yuav tsis pub kom kouvlub npe thiab lwm cov ntaub ntawv qhia tau lwm tus neeg sab nraud kom taug qab txog tau kuv

_________ ___________ Kawm txog cov tshuaj uas koj noj puas txhaum los tsis txhaum rau caj ceg
_________ ___________ Kawm txog saib seb kab mob thiab caj ceg puas txhaum los tsis txhaum

Thov kos npe yog tias koj pom zoo koom tes rau qhov kev kawm tshawb fawb no. Peb mam li luam ib daim ntawv no rau koj thiab.

____ __________________
Kos npe/tus neeg yuav koom txoj kev kawm tshawb fawb no   Hnub Tim(Hli,hnub/xyoo)

____
Sau npe/tus neeg yuav koom txoj kev kawm tshawb fawb no

____ __________________
Kos npe/kws tshawb fawb   Hnub Tim(Hli,hnub/xyoo)

____
Sau npe/kws tshawb fawb

WITNESS STATEMENT:

Tus neeg ua pov thawj:

Tus neeg koom qhov kev kawm tshawb fawb no sau thiab kos tsis tau lawv lub npe rau daim ntawv tso cai no vim hais tias:
☐ The participant is illiterate

Tus neeg no tsis txawj ntaub ntawv
☐ The participant is visually impaired

Tus neeg no dig muag los yog qhov muag tsis pom zoo
☐ The participant is physically unable to sign the consent form.
Describe:___________________________

Tus neeg no siv tsis tau lub zog los kos npe rau daim ntawv no. Qhia vim li cas:__________________________
☐ Other *(please specify)*: ____

Muaj lwm yam (thov qhia):___________________________________

Qhov no yog rau tus neeg ua hawjlwm txhais lus:
Kuv tau taub lus Aaskiv thiab cov lus uas tus neeg koom qhov kev kawm tshawb fawb hais. Kuv lees hais tias cov lus Aaskiv ntawm daim ntawv no tau muab txhais los hais rau tus neeg uas koom qhov kev kawm tshawb fawb no kom nws tau taub raws lis cov lus uas nws paub. Tus kws tshawb fawb yeej cia nws hais nws tej lus nug thiab.

____ __________________
Kos Npe/Tus neeg txhais lus Hnub Tim(Hli,hnub/xyoo)

____
Sau Npe/Tus neeg txhais lus

OR: Statement from a Non-Interpreter:
Nov yog rau tus neeg txhais lus uas tsis yog neeg ua haujlwm:

Kuv tau taub lus Aaskiv thiab cov lus uas tus neeg koom qhov kev tshawb fawb hais. Kuv lav hais tias cov lus Aaskiv ntawm daim ntawv no tau muab txhais los hais rau tus neeg uas koom qhov kev kawm tshawb fawb no kom nws tau taub raws lis cov lus uas nws paub. Tus kws tshawb fawb yeej cia nws hais nws tej lus nug thiab.

____ __________________
Kos Npe/Tus neeg txhais lus Hnub Tim(Hli,hnub/xyoo)
____
Sau Npe/Tus neeg txhais lus
