# Supplementary material for: Hmong microbiome ANd Gout, Obesity, Vitamin C (HMANGO-C): A phase II clinical study protocol
Source: PLoS One. 2023 Feb 1;18(2):e0279830. doi: 10.1371/journal.pone.0279830 (PMC9891498; doi:10.1371/journal.pone.0279830)
Supplement: S5 File — (PDF) [file pone.0279830.s006.pdf]

University of Minnesota

TSO CAI LOS SIV COV NTAUB NTAWV KHO MOB RAU KEV TSHAWB FAWB HIPAA[1] DAIM FOOS TSO CAI

IRB Tus Naj Npawb Kawm Ntawv: **Nyem los sis kov no mus rau kev sau cov ntaw:**
Lub Npe Kev Kawm: **Nyem los sis kov no mus rau kev sau cov ntaw:**
Tus Tuam Thawj Saib Xyuas Tsev Kawm Npe: **Nyem los sis kov no mus rau kev sau cov ntaw:**
Tus Thawj Saib Xyuas Tsev Kawm Ntawv Qhov Chaw Nyob: **Nyem los sis kov no mus rau kev sau cov ntaw:**

A. Lub hom phiaj ntawm daim foos no yog dab tsi?

Lub hom phiaj ntawm daim foos no yog koj tso cai rau peb siv thiab qhia tawm koj cov ntaub ntawv kev noj qab haus huv rau cov kev tshawb fawb uas sau cia saum toj no, thiab yog tias peb xav tau koj cov ntaub ntawv kho mob, muab kev tso cai rau cov kws saib xyuas kev noj qab haus huv uas kho koj kom faib koj cov ntaub ntawv khob mob rau peb rau kev tshawb fawb. Lub hom phiaj ntawm daim foos no yog koj tso cai rau peb siv thiab qhia tawm koj cov ntaub ntawv kev noj qab haus huv rau cov kev tshawb fawb tshawb fawb teev saum toj no, thiab yog tias peb xav tau koj cov ntaub ntawv kho mob, muab kev tso cai rau cov kws saib xyuas kev noj qab haus huv uas kho koj kom faib koj cov tshuaj cov ntaub ntawv nrog peb tshawb fawb. Daim foos no tseem piav qhia txog cov ntaub ntawv kev noj qab haus huv uas yuav siv rau kev tshawb fawb. Yog koj txiav txim siab tso cai thiab koom tes nrog rau kev tshawb fawb, koj yuav tsum kos npe rau daim foos no thiab Daim Ntawv Pom Zoo. Koj yuav tsum paub tias thaum koj cov ntaub ntawv kev noj qab haus huv raug faib rau lwm tus neeg raws li tau piav qhia hauv daim foos no, nws yuav tsis raug tiv thaiv los ntawm txoj cai ntiag tug, thiab yuav muab rau lwm tus tshaj li cov tau hais tseg hauv daim foos no los sis Daim Ntawv Tso Cai.

B. Cov ntaub ntawv xov xwm kho mob twg yuav ua rau muaj?

Cov ntaub ntawv kev noj qab haus huv hais txog koj los siv thiab qhia tawm rau kev tshawb fawb suav nrog cov khoom uas tshawb xyuas los ntawm pab neeg tshawb fawb hauv qab no:

☐Koj cov ntaub ntawv teev kho mob, uas yuav suav nrog cov ntaub ntawv los ntawm tsev kho mob thiab chaw kho mob, mus ntsib chav kho mob ceev, tshuaj tiv thaiv, keeb kwm kev kho mob thiab kuaj lub cev, tshuaj, duab thiab ntawv tshaj tawm, sau ntawv qhia kev nruam ntej, kev ntsuam xyuas mob hlwb, EEG / EKG / ECHO cov ntawv tshaj tawm, kev kuaj ntshav thiab kab mob, cov ntaub ntawv kho hniav thiab cov ntaub ntawv nyiaj txiag. Cov ntaub ntawv no yuav siv thiab qhia tawm kom ntev li ntev yog txoj kev tshawb fawb muaj txuas ntxiv.

---

[1] HIPAA yog Txoj Cai Pab Cuam Kev Noj Qab Haus Huv Kom Zoo thiab Kev Ris Dej Num ntawm 1996, tsoom fwv txoj cai lij choj ntsig txog kev ceev ntiag tug ntawm cov ntaub ntawv kev noj qab haus huv.

☒Cov ntaub ntawv khaws cia ua ib feem ntawm qhov kev tshawb fawb no, suav nrog cov txheej txheem tshawb nrhiav, kev mus ntsib kev tshawb nrhiav, thiab txhua yam kev xaiv ntawm kev tshawb pom uas koj pom zoo, txhua yam tau piav nyob rau hauv Daim Ntawv Tso Cai. Cov ntaub ntawv no tej zaum yuav tsis yog ib feem ntawm koj cov ntaub ntawv teev kev kho mob, thiab tej zaum suav nrog kev teb rau cov soj ntsuam thiab cov lus nug, thiab cov ntaub ntawv khaws thaum lub sij hawm mus ntsib kev tshawb fawb piav nyob rau hauv Daim Ntawv Tso Cai.

C. Hais txog cov ntaub ntawv kho mob hais txog tej yam tob tob ne yuav ua li cas?

Qee cov ntaub ntawv kev noj qab haus huv hais txog tej yam tob tob uas nws yuav tsum tau txais koj kev tso cai tshwj xeeb. Yog tias qhov kev kawm tshawb fawb koj tau koom nrog yuav tsum tau ib qho ntawm cov ntaub ntawv hais txog tej yam tob tob no, cov npov hauv qab no yuav raug cim thiab koj yuav raug nug kom kos npe luv tso cai rau cov ntaub ntawv yuav tsum muaj rau pawg neeg tshawb fawb siv thiab sib qhia raws li tau piav hauv Daim Ntawv Tso Cai Cov.

☐ Kuv li ntaub ntawv qhiav txog kev quav yeeb tshuaj thiab dej caw, kuaj mob thiab ntaub ntawv teev txog kev kho mob. ____ (kos npe luv)
☐ Kuv cov ntaub ntawv kuaj kab mob HIV / AIDS .____ (kos npe luv)
☐ Kuv cov ntaub ntawv qhia txog caj ces ._____ (kos npe luv)
☐ Kuv cov ntaub ntawv kuaj mob los sis kho mob hlwb ._____ (kos npe luv)
☐ Kuv cov ntawv teev ntshav muaj mob ntshav qis ._____ (kos npe luv)

D. Leej twg yuav nkag thiab siv kuv cov ntaub ntawv kev noj qab haus huv?

Yog koj pom zoo koom nrog txoj kev tshawb fawb no, koj cov ntaub ntawv kev noj qab haus huv yuav raug muab qhia rau:

1. Pawg neeg tshawb fawb uas ua qhov kev tshawb fawb tau piav qhia nyob rau hauv Daim Ntawv Tso Cai Pom Zoo, suav nrog txhua lub koom haum koom tes los sis cov tsev kawm txog kev tshawb fawb uas muaj npe koom uas tau piav qhia hauv Daim Ntawv Tso Cai Pom Zoo;

2. Lwm cov neeg hauv University of Minnesota and M Health/Fairview uas pab txhawb rau cov kev tshawb fawb los sis uas yog tus saib xyuas kev tshawb fawb (xws li Pawg Tuam Thawj Saib Xyuas Kev Tshawb Fawb los sis IRB uas yog pawg kws saib xyuas thiab muab kev saib xyuas sab kev coj zoo thiab kev ua raws kev cai li choj ntawm kev tshawb fawb hauv University, cov thawj tswj hauj lwm thiab lwm yam cov neeg ua hauj lwm muab kev pab sab txuj ci thiab / los sis kev tswj hwm, kev ua kom raws cai thiab kev kuaj xyuas yam tshaj li, cov neeg uas koom nrog kev cov txheej txheem them nyiaj uas koj yuav tau txais rau koj kev koom tes, thiab lwm yam);

3. Tus (cov) kws tshawb fawb , txhua cov kev koom raws cai, cov koom tes los sis cov sawv cev ntawm tus (cov) neeg pab txhawb nqa uas koom nrog txoj kev tshawb fawb, cov koom haum pab nyiaj rau txoj kev tshawb fawb, thiab txhua tus uas koom nrog, cov kev koom raws cai los sis cov chaw sawv cev ntawm lub (cov) koom haum pab nyiaj uas koom nrog kev tshawb fawb;

4. Cov koom haum uas muab kev lees paub thiab kev saib xyuas rau pab neeg tshawb fawb, thiab lwm tus uas tau kev tso cai los ntawm kev cai li choj los saib xyuas qhov zoo thiab kev nyab xeeb ntawm kev tshawb fawb (xws li Asmeskas tsoom fwv cov koom haum xws li Kev Tshwj Fwm Zaub Mov thiab Tshuaj, Lub Chaw Hauj Lwm ntawm Kev Tiv Thaiv Tib Neeg Kev Tshawb Fawb, Lub Chaw Hauj Lwm Kev Tshawb Fawb Kev Muaj Siab Dawb Paug, los sis tsoom fwv cov koom haum hauv lwm cov tebchaws); thiab

5. Cov koom haum ua cov txheej txheem them nyiaj uas yuav muab rau koj rau kev koom nrog qhov kev kawm no thiab lwm tus neeg los sis cov koom haum uas tau teev tseg hauv Daim Ntawv Tso Cai Pom Zoo.

E. **Kuv puas yuav raug nqua hu tias yuav tsum kos npe rau daim foos no?**

Tsis, koj tsis raug nqua hu tias yuav tsum kos npe rau daim foos no. Txawm li cas los xij, yog tias koj tsis kos npe rau daim foos no, koj yuav tsis muaj feem koom nrog kev kawm tshawb fawb no. Kev kho mob muaj nyob rau sab nraud ntawm txoj kev tshawb, kev them nyiaj rau kev kho mob, kev sau npe hauv cov phiaj xwm kev noj qab haus huv thiab kev tsim nyog tau txais txiaj ntsig yuav tsis cuam tshuam dab tsi los ntawm koj qhov kev txiav txim siab kos npe rau daim foos no.

F. **Kuv puas yuav tuaj yeem saib tau kuv cov ntaub ntawv?**

Nws nws kuj muaj tau tias pawg neeg tshawb fawb yuav tsis tso cai rau koj kom pom cov ntaub ntawv khaws tseg rau qhov kev tshawb fawb no. Txawm li cas los xij, koj tuaj yeem nkag mus saib cov ntaub ntawv tso rau hauv koj cov ntawv kho mob tom qab kev tshawb fawb tiav.

G. Kev xaiv kev tshawb fawb kev ua ub no

Qhov kev kawm tshawb fawb uas koj koom nrog tej zaum yuav muaj cov kev xaiv tshawb fawb kev ua ub ua no los cuam tshuam nrog nws, txhais tau tias koj tsis tas yuav pom zoo rau cov kev ua hub ua no no kom koj tuaj yeem koom nrog qhov kev tshawb fawb kev kawm paub no. Thov qhia koj txoj kev txaus siab koom nrog cov kev xaiv ua ub ua no no thiab tso cai siv koj cov ntaub ntawv los ntawm kev xaiv kev ua ub ua no no raws li tau piav qhia hauv qab no los ntawm qhov koj sau koj lub npe luv rau ntawm ib sab.

Tso Cai

Koj xav teb cov lus nug no thiab tsis xav teb los tau

Cov lus nug no yog rau koj teb thiab tsis teb los nyob ntawm koj. Qhov nov tsis cuam tshuam txog qhov koj los koom qhov kev kawm tshawb fawb no. Thov kos koj lub npe hauv qab no qhov Tso Cai, los qhov Tsis Tso Cai.

**Yog lawm, Kuv pom zoo**	**Tsis yog, Kuv tsis pom zoo**	
____________	____________	Kws tshawb fawb ntaus ntawv hauv xov tooj tuaj rau kuv txog nyiaj hauv koj daim Greenphire Clincard.
____________	____________	Kws tshawb fawb nrog kuv tus kws kho mob tham txog kuv cov tshuaj uas kuv noj lub sijhawm no.
____________	____________	Kws tshawb fawb yuav hu rau kuv txog lwm qhov kev kawm tshawb fawb yav tom ntej.
		Kws tshawb fawb siv tau kuv cov quav thiab caj ceg roj ntshav tom qab qhov kev kawm tshawj fawb no xaus. Yuav tsis pub kom kouvlub npe thiab lwm cov ntaub ntawv qhia tau lwm tus neeg sab nraud kom taug qab txog tau kuv
____________	____________	Kawm txog cov tshuaj uas koj noj puas txhaum los tsis txhaum rau caj ceg
____________	____________	Kawm txog saib seb kab mob thiab caj ceg puas txhaum los tsis txhaum

H. **Kuv txoj kev pom zoo rau kev muab kuv cov ntaub ntawv teev kho mob rau kev siv thiab qhia tawm puas txawj tas sij hawm?**

Tsis, tsis muaj hnub tas sij hawm.

I. **Kuv puas tuaj yeem thim kuv qhov kev tso cai rau kev siv thiab qhia tawm ntawm kuv cov ntaub ntawv teev kev noj qab haus huv?**

Yog lawm. Koj tuaj yeem thim koj txoj kev tso cai lub sij hawm twg los tau los ntawm kev sau ntawv mus rau tus kws tshawb fawb ntawm qhov chaw nyob uas nyob saum toj ntawm daim ntawv no. Yog tias koj thim koj qhov kev tso cai, koj yuav tsis nyob hauv qhov kev tshawb fawb ntxiv lawm. Koj yuav tau nug ib tug neeg hauv pab pawg kev tshawb fawb yog tias kev rho tawm yuav cuam tshuam rau kev tshawb fawb txog kev kho mob. Yog tias koj tshem tawm koj qhov kev tso cai, txhua cov ntaub ntawv kev noj qab haus huv txog koj uas twb siv thiab qhia tawm tuaj yeem siv txuas ntxiv rau kev tshawb fawb thiab lwm qhov kev xaiv rau kev kawm uas koj tau pom zoo saum toj no.

J. Kos Npe

Yog koj pom zoo rau kev siv thiab qhia tawm koj cov ntaub ntawv kev noj qab haus huv raws li tau piav qhia hauv daim ntawv no, thov sau koj lub npe thiab kos npe rau hauv qab no. Koj yuav tau txais ib daim qauv foos uas koj tau kos npe no**.**

____

Tus Koom Tes Kev Tshawb Fawb Lub Npe (luam tawm)
(*yuav tsum tau ua txawm tias raug kos npe los ntawm niam thiab txiv / tus sawv cev raws cai*)

____ __________________

Tshawb Fawb Tus Neeg Koom Tes Kos Npe Hnub Tim
(yuav tsum tau ua txawm tias daim foos no raug nyeem rau tus neeg koom vim tias nws nyeem tsis tau daim foos)

<u>Niam Txiv los sis Tus Sawv Cev Tso Cai</u>

Yog tias koj pom zoo rau kev siv thiab tso tawm Cov Ntaub Ntawv Qhia Txog Kev Noj Qab Haus Huv ntawm Tus Neeg Koom Kev Tshawb Fawb muaj npe saum toj no, thov luam koj lub npe thiab kos npe hauv qab.

____

Niam thiab Txiv los sis Tus Sawv Cev Tso Cai Raug Cai lub npe (luam tawm)

____

Kev Sib Raug Zoo Nrog Tus Neeg Tuaj Koom Kev Tshawb Fawb

____ __________________

Niam thiab Txiv los sis Tus Sawv Cev Tso Cai Raug Cai Kos Npe Hnub Tim

Tus Neeg Pov Thawj / Neeg Txhais Lus

Kuv kos npe rau hauv qab no ua pov thawj tias cov ntaub ntawv hauv HIPAA Daim Ntawv Tso Cai tau raug piav qhia meej (los yog nyeem) rau, thiab pom tau tias nkag siab los ntawm, tus neeg koom, thiab qhov kev tso cai tau txais los ntawm tus neeg koom yam tsis muaj kev quab yuam.

____

Tus Ua Pov Thawj / Tus Txhais Lus Npe (sau)

____ __________________

Tus Neeg Pom / Neeg Txhais Lus Kos Npe Hnub Tim

Instructions for Researchers: <u>Do not make any changes</u> to this form other than the following items:

The IRB **will not** be confirming the accuracy of the information you complete on this form. The researchers are responsible for accurately completing the HIPAA Research Authorization as follows:

1. Section B: Mark all sources of health information that will be released to the research team from M Health or other providers.

2. Section C:
   a. Check the box ***only*** for each specific type of information that will be collected for this study
   b. Records for drug and alcohol abuse, diagnosis and treatment are records related to admissions to treatment centers; records for mental health diagnosis or treatment are records related to admissions to mental health units
   c. Obtain the participant's initials ***only*** *for the specific types of information checked*

3. Section G:
   a. Check the boxes indicating if there are optional research activities or not
   b. Obtain the participant's initial *only if the study involves optional research activity*

4. Section J: Obtain the participant's name, signature, and date; *complete subsequent signature lines* ***if applicable***

5. Provide the participant with a signed copy of the form

Note: This form allows you to check the boxes electronically. You can make a 'master version' of this form for this study with all pertinent boxes checked.
