# Supplementary material for: Hmong microbiome ANd Gout, Obesity, Vitamin C (HMANGO-C): A phase II clinical study protocol
Source: PLoS One. 2023 Feb 1;18(2):e0279830. doi: 10.1371/journal.pone.0279830 (PMC9891498; doi:10.1371/journal.pone.0279830)
Supplement: S6 File — (PDF) [file pone.0279830.s007.pdf]

DO YOU HAVE GOUT?

OR EVEN IF YOU DON'T...

JOIN THE RESEARCH PROJECT CALLED HMANGO-C

HMONG MICROBIOME AND GOUT, OBESITY, VITAMIN C

THIS RESEARCH COULD HELP the Hmong community learn more about gout and ways to treat it

Join the study and help our community learn:

- If vitamin C lowers uric acid and gout symptoms
- Why some Hmong people have gout and other people don't
- Why some foods cause gout attacks and other foods don't
- If germs in stool (microbiome) affect uric acid and obesity
- If some genes (DNA) affect uric acid

Interested? Contact Toua Yang, Yeng Moua or Bai Vue in Hmong or English

Phone: (612) 440-4170

Email: hmangoc2020@gmail.com

Website: hmangoc.org

Researchers:
Hmong Gout Coalition
University of Minnesota
SoLaHmo Partnership for health and wellness

THIS RESEARCH IS FOR...

1. Hmong adults
2. With or without gout
3. Live close to Mpls/St Paul, MN, USA

YOU WILL...

1. Answer questions about diet and health
2. Give blood, urine, spit, and stool
3. Take Vitamin C tablets twice a day for 8 weeks
4. Get up to $150.00

You, your family, and your friends are invited to join the HMANGO-C study!

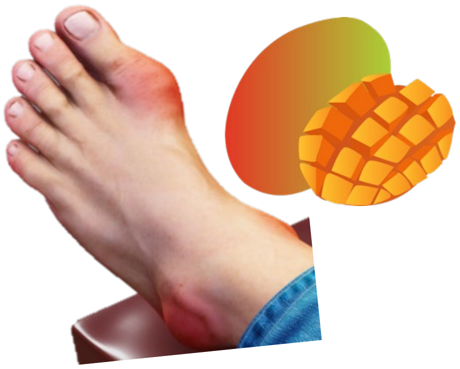

UMN IRB Approval #_STUDY00010406_
Approved Date _1/26/2021_
