# Supplementary material for: Hmong microbiome ANd Gout, Obesity, Vitamin C (HMANGO-C): A phase II clinical study protocol
Source: PLoS One. 2023 Feb 1;18(2):e0279830. doi: 10.1371/journal.pone.0279830 (PMC9891498; doi:10.1371/journal.pone.0279830)
Supplement: S7 File — (PDF) [file pone.0279830.s008.pdf]

Koj puas mob ko taw vwm?

TXAWM KOJ TSIS MOB LOS...

TUAJ KOOM PEB QHOV KEV TSHAWB FAWB UAS MUAJ LUB NPE HU UA HMANGO-C, HMONG MICROBIOME AND GOUT, OBESITY, VITAMIN C

QHOV KEV TSHAWB FAWB NO PAB TAU peb Hmoob kawm txog tus mob ko taw vwm thiab xav nrhiav tshuaj pab.

Tuaj koom qhov kev tshawb fawb no thiab pab peb haiv neeg Hmoob kawm txog:

- Saib seb vitamin C puas tua tau tus kab mob ko taw vwm thiab saib seb puas pab tau tsos mob ko taw vwm
- Ua cas Hmoob ho mob ko taw vwm ntau dua lwm haiv neeg
- Vim li cas ib co zaub mov ua tau tus mob ko taw vwm huam tuaj ntau dua lwm cov
- Saib seb yus cov quav qhia tau li cas txog yus cov ntshav mob ko taw vwm thiab qhov yus rog
- Saib yus cov roj ntsha puas qhia tau li cas txog cov ntshav mob ko taw vwm

Yog koj xav paub ntxiv, hu tau rau: Toua Yang, Yeng Moua los yog Bai Vue
Tham tau lus Hmoob thiab Aaskiv
Phone: (612) 440-4170
Email: hmangoc2020@gmail.com
Website: hmangoc.org

Koom haum tshawb fawb:
Hmong Gout Coalition
University of Minnesota
SoLaHmo Partnership for health and wellness

QHOV KEV TSHAWB FAWB YOG RAU...

1. Hmoob 18 xyoo rov sauv
2. Muaj mob ko taw vwm los yog tsis mob yeej tau
3. Nyob ze Mpls/St Paul, MN, USA

KOJ YUAV...

1. Tau teb lus nug txog kev noj haus thiab kev noj qab haus huv
2. Kuaj koj cov ntshav, zis, qaub ncaug, quav
3. Txhua hnub noj ob ntsiav Vitamin C kom txog 8 lub lim tiam
4. Tau nyiaj txog $150.00

Koj, koj tsev neeg, thiab phooj ywg koom tau qhov kev tshawb fawb HMANGO-C

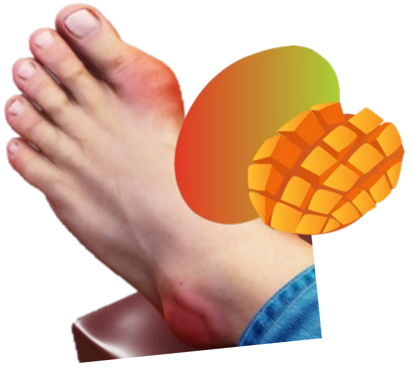

UMN IRB Approval # STUDY00010406
Approved Date 1/26/2021
