# Supplementary material for: Hmong microbiome ANd Gout, Obesity, Vitamin C (HMANGO-C): A phase II clinical study protocol
Source: PLoS One. 2023 Feb 1;18(2):e0279830. doi: 10.1371/journal.pone.0279830 (PMC9891498; doi:10.1371/journal.pone.0279830)
Supplement: S8 File — (PDF) [file pone.0279830.s009.pdf]

GAQ2.0

REVISED GOUT IMPACT (GI) SECTION OF GAQ2.0

Reprinted with permission. Copyright © 2008 Takeda Global Research & Development Inc.

Please answer every question. Read every question carefully and choose the best answer for you.
Question may be answered by filling in a bubble to indicate your choice.
Some questions in this survey are about your gout overall and some are about only the times you are experiencing pain or swelling of your joints due to your gout. Tow important terms are used in this survey:

***Gout Attack* =** time when you are experiencing pain or swelling of your joints because of gout. When a question is about a Gout Attack, please only think about what it is like for you when you have joint pain or swelling because of your gout.

***Gout Overall* =** times you have a Gout Attack AND the time Between Attacks when you do not have joint pain or swelling because of gout

Medication=prescribed medications from doctors

**Item**
1. Mob ko taw vwm ntawm koj lub neej txhua hnub zoo li cas?<br><br>Xaiv saib seb cov lus nug no puas raug li koj siab xav.
a. Kuv txhawj hais tias kuv yuav mob ko taw vwm xyoo no.
b. Kuv ntshai tias kuv tus mob ko taw vwm yuav huam loj tuaj yav tom ntej no.
c. Kuv txhawj xeeb tias kuv tus mob ko taw vwm yuav cuam tshuam txog lwm yam hauv kuv lub neej tom ntej no.
d. Kuv txhawj tias kuv yuav ua tsis tau tej yam lom zem raws li kuv siab xav vim kuv mob ko taw vwm.
e. Kuv tsis noj kuv cov tshuaj mob ko taw vwm uas kws kho mob muab vim tsis haum kuv.
f. Thaum kuv mob ko tau vwm, kuv chim siab.
g. Nws yeej nyuaj thaum yuav npaj ua dabtsis vim kuv txhawj tias kuv yuav mob ko taw vwm.
h. Kuv yeej nyuaj siab thaum kuv mob ko taw vwm.
i. Tamsis no, kuv yeej muaj tshuaj loog zoo los ntawm kws kho mob rau thaum kuv mob ko taw vwm.
j. Txawm kuv tseem npaj ua dabtsis los kuv yeej ua tsis tau yog thaum kuv mob ko taw vwm.
k. Kuv txhawj hais tias kuv cov tshuaj los ntawm kws kho mob yuav tsis zoo rau kuv ntev mus.
l. Cov tshuaj los ntawm kws kho mob tamsis no tiv thaiv tsis tau kom kuv txhob mob ko taw vwm.
m. Kuv yeej tswj tau kuv tus mob ko taw vwm.
2. Thaum koj mob ko taw vwm tag los no, nws cuam tshuam cov no li cas?
a. Qhaj hawjlwm thaum mob ko taw vwm?
b. Ua hawjlwm nyuaj nyuaj thaum mob ko taw vwm?
c. Tawm rooj los yog mus uasi nyuaj nyuaj thaum mob ko taw vwm?
d. Tu koj tus kheej li nyuaj nyuaj thaum mob ko taw vwm, xws li noj mov, da dej, los yog hnav ris tsho?

3. Thaum koj mob ko taw vwm tag los no, nws cuam tshuam cov no li cas?
a. Koj txoj kev xav, kev nyuaj siab thiab kev zoo siab?
b. Koj txoj kev txav tau yoojyim?
c. Lub sijhawm pw?
d. Ua hawjlwm li qub?
e. Koj txoj kev uasi?
f. Koj txoj kev nyob ywj siab?
g. Your ability to do what you want to do?<br>Koj txoj kev ua tau raws li koj xav?
h. Your social obligations?<br>Koj tes dej num rau koj tsev neeg thiab kwv tij neej tsa?
Scales and items: Gout concern overall (4 items, 1 a-d); Gout medication side effects (2 items, 1 e & K); Unmet gout treatment need (3 items, 1 i,l,m); Well being during attack (11 items, 2 a-d 3 a-g); Gout concern during attack (4 items, 1 f,g,h,j)
