# Supplementary material for: Hmong microbiome ANd Gout, Obesity, Vitamin C (HMANGO-C): A phase II clinical study protocol
Source: PLoS One. 2023 Feb 1;18(2):e0279830. doi: 10.1371/journal.pone.0279830 (PMC9891498; doi:10.1371/journal.pone.0279830)
Supplement: S9 File — (PDF) [file pone.0279830.s010.pdf]

HMANGO-C Study
Vitamin C Daily Log

April 2022

Sun	Mon	Tue	Wed	Thu	Fri	Sat
					01<br>____ AM PM*<br>____ AM PM	02<br>____ AM PM<br>____ AM PM
03<br>____ AM PM<br>____ AM PM	04<br>____ AM PM<br>____ AM PM	05<br>____ AM PM<br>____ AM PM	06<br>____ AM PM<br>____ AM PM	07<br>____ AM PM<br>____ AM PM	08<br>____ AM PM<br>____ AM PM	09<br>____ AM PM<br>____ AM PM
10<br>____ AM PM<br>____ AM PM	11<br>____ AM PM<br>____ AM PM	12<br>____ AM PM<br>____ AM PM	13<br>____ AM PM<br>____ AM PM	14<br>____ AM PM<br>____ AM PM	15<br>____ AM PM<br>____ AM PM	16<br>____ AM PM<br>____ AM PM
17<br>____ AM PM<br>____ AM PM	18<br>____ AM PM<br>____ AM PM	19<br>____ AM PM<br>____ AM PM	20<br>____ AM PM<br>____ AM PM	21<br>____ AM PM<br>____ AM PM	22<br>____ AM PM<br>____ AM PM	23<br>____ AM PM<br>____ AM PM
24<br>____ AM PM<br>____ AM PM	25<br>____ AM PM<br>____ AM PM	26<br>____ AM PM<br>____ AM PM	27<br>____ AM PM<br>____ AM PM	28<br>____ AM PM<br>____ AM PM	29<br>____ AM PM<br>____ AM PM	30<br>____ AM PM<br>____ AM PM

*Tell us when in the day you took the two vitamin doses like
_8_ (AM) PM
_6_ AM (PM)
