# Supplementary material for: Hmong microbiome ANd Gout, Obesity, Vitamin C (HMANGO-C): A phase II clinical study protocol
Source: PLoS One. 2023 Feb 1;18(2):e0279830. doi: 10.1371/journal.pone.0279830 (PMC9891498; doi:10.1371/journal.pone.0279830)
Supplement: Attachment — Submitted filename: Response to Reviewers_09.15.2022.docx [file pone.0279830.s011.docx]

September 15, 2022

Re: Response to reviewer’s comments. (Manuscript ID PONE-D-22-04805)

Thank you for your review of our research protocol “Hmong Microbiome ANd Gout, Obesity, Vitamin C (HMANGO-C): A phase II clinical study protocol”.

We appreciated the insightful review from the reviewers regarding the above article. We are delighted to be able to respond to each of the comments and believe our responses address concerns and comments of the referees.

The following includes our responses to the specific comments made and identifies changes made to the current version of the manuscript.

**Journal requirements:**

1. Please ensure that your manuscript meets PLOS ONE's style requirements, including those for file naming. The PLOS ONE style templates can be found at

https://journals.plos.org/plosone/s/file?id=wjVg/PLOSOne_formatting_sample_main_body.pdf and

https://journals.plos.org/plosone/s/file?id=ba62/PLOSOne_formatting_sample_title_authors_affiliations.pdf".

**Response**: The title/authors/affiliation page and main body manuscript have been modified according to the PLOS ONE's style requirements.

2. Thank you for stating the following in the Funding Section of your manuscript:

“Minnesota Partnership for Biotechnology and Medical Genomics grant #18.08, Targeting the gut microbiome to prevent the increasing incidence of obesity in immigrant populations. This funding source had no role in the design of this study and will not have any role during its execution, analyses, interpretation of the data or decision to submit results.”

We note that you have provided funding information that is not currently declared in your Funding Statement. However, funding information should not appear in the Acknowledgments section or other areas of your manuscript. We will only publish funding information present in the Funding Statement section of the online submission form.

Please remove any funding-related text from the manuscript and let us know how you would like to update your Funding Statement. Currently, your Funding Statement reads as follows:

“D.K received funding from Minnesota Partnership for Biotechnology and Medical Genomics grant #18.08, Targeting the gut microbiome to prevent the increasing incidence of obesity in immigrant populations. This funding source had no role in the design of this study and will not have any role during its execution, analyses, interpretation of the data or decision to submit results.”

Please include your amended statements within your cover letter; we will change the online submission form on your behalf.

**Response**: Please revise the Funding statement is as follows:

*D.K. received funding from Minnesota Partnership for Biotechnology and Medical Genomics grant #18.08, Targeting the gut microbiome to prevent the increasing incidence of obesity in immigrant populations. This funding source had no role in the design of this study and will not have any role during its execution, analyses, interpretation of the data or decision to submit results. This study was also supported by the University of Minnesota Clinical and Translational Science Institute, the Graduate School at the University of Minnesota, University of Minnesota and National Institutes of Health’s National Center for Advancing Translational Sciences, grant UL1TR002494. The content is solely the responsibility of the authors and does not represent the official views of the National Institutes of Health’s National Center for Advancing Translational Sciences.*

3. Thank you for stating the following in the Competing Interests section:

“D.K. serves as CEO and holds equity in CoreBiome, a company involved in the commercialization of microbiome analysis. The University of Minnesota also has financial interests in CoreBiome under the terms of a license agreement with CoreBiome. These interests have been reviewed and managed by the University of Minnesota in accordance with its Conflict-of-Interest policies.”

Please confirm that this does not alter your adherence to all PLOS ONE policies on sharing data and materials, by including the following statement: "This does not alter our adherence to PLOS ONE policies on sharing data and materials.” (as detailed online in our guide for authors http://journals.plos.org/plosone/s/competing-interests). If there are restrictions on sharing of data and/or materials, please state these. Please note that we cannot proceed with consideration of your article until this information has been declared.

Please include your updated Competing Interests statement in your cover letter; we will change the online submission form on your behalf.

**Response**: Please revise the Competing Interests statement is as follows:

*D.K. serves as CEO and holds equity in CoreBiome, a company involved in the commercialization of microbiome analysis. The University of Minnesota also has financial interests in CoreBiome under the terms of a license agreement with CoreBiome. These interests have been reviewed and managed by the University of Minnesota in accordance with its Conflict-of-Interest policies. This does not alter our adherence to PLOS ONE policies on sharing data and materials.*

4. In your Data Availability statement, you have not specified where the minimal data set underlying the results described in your manuscript can be found. PLOS defines a study's minimal data set as the underlying data used to reach the conclusions drawn in the manuscript and any additional data required to replicate the reported study findings in their entirety. All PLOS journals require that the minimal data set be made fully available. For more information about our data policy, please see http://journals.plos.org/plosone/s/data-availability.

Upon re-submitting your revised manuscript, please upload your study’s minimal underlying data set as either Supporting Information files or to a stable, public repository and include the relevant URLs, DOIs, or accession numbers within your revised cover letter. For a list of acceptable repositories, please see http://journals.plos.org/plosone/s/data-availability#loc-recommended-repositories. Any potentially identifying patient information must be fully anonymized.

Important: If there are ethical or legal restrictions to sharing your data publicly, please explain these restrictions in detail. Please see our guidelines for more information on what we consider unacceptable restrictions to publicly sharing data: http://journals.plos.org/plosone/s/data-availability#loc-unacceptable-data-access-restrictions. Note that it is not acceptable for the authors to be the sole named individuals responsible for ensuring data access.

We will update your Data Availability statement to reflect the information you provide in your cover letter.

**Response**: At this time, we do not have research data available. However, we are committed to share the de-identified minimal data set and the data sharing plan is stated in the Data Availability statement. Updated Data Availability statement is as follows:

*De-identified relevant clinical data will be made available upon study completion. The minimal data set, including shotgun metagenomic sequencing data will be deposited in GenBank®, the National Institute of Health genetic sequence database.*

5. We note that the original protocol file you uploaded contains a confidentiality notice indicating that the protocol may not be shared publicly or be published. Please note, however, that the PLOS Editorial Policy requires that the original protocol be published alongside your manuscript in the event of acceptance. Please note that should your paper be accepted, all content including the protocol will be published under the Creative Commons Attribution (CC BY) 4.0 license, which means that it will be freely available online, and any third party is permitted to access, download, copy, distribute, and use these materials in any way, even commercially, with proper attribution.

Therefore, we ask that you please seek permission from the study sponsor or body imposing the restriction on sharing this document to publish this protocol under CC BY 4.0 if your work is accepted. We kindly ask that you upload a formal statement signed by an institutional representative clarifying whether you will be able to comply with this policy. Additionally, please upload a clean copy of the protocol with the confidentiality notice (and any copyrighted institutional logos or signatures) removed.

**Response**: The confidentiality mentioned in the study protocol is pertaining to the data security of the study participants. There are no restrictions regarding the sharing of our study protocol with the public.

**Reviewer 1**

This is an interesting and well written paper; the protocol is carefully described.

Some comments.

In the introduction the possible role of Vit C on gut microbiota should be shortly added to better define point 1 of the secondary objective, e.g. in a healthy population high doses of Vit C have shown a positive shift of bacteria populations in the gut (Otten 2021).

**Response**: Further information on the comparison of gut microbiota between healthy adults and patients with gout is now provided. The impact of urate-lowering therapy such as allopurinol and vitamin C on gut microbiota is also included in the introduction section.

Similarly, in the introduction when dealing with metabolic diseases, published data on the presence of dysbiosis in gout patients and the role of specific urate-reducing drugs in modifying gut microbiota should be provided.

**Response**: Please see the response as above.

Are pts under diuretic treatment allowed in the study?

**Response**: Yes, patients on diuretic treatments are allowed in the study. We plan to adjust relevant concomitant medications, including the diuretics, in the analysis as stated in the “Analysis Plan” section.

"To assess study medication adherence, we used the medication diary sheet recorded by participants at Visit 2". It should be discussed that the assessment of blood levels of ascorbic acid after treatment is not planned.

**Response**: A clarification sentence is added to address this point.

“The assessment of blood levels of ascorbic acid after treatment to verify the medication adherence is not planned because ascorbic acid is rapidly degraded [50](Karlsen 2007) and current study design did not allow a reliable measurement of ascorbic acid.”

**Reviewer 2**

This study protocol is well structured and well elaborated., although there are some critical points that the authors should remedy.

The authors should better explain the rationale for the administration of vitamin C to subjects without gout/hyperuricaemia.

**Response**: As described in the background, a previous meta-analysis had demonstrated at a median dose of vitamin C 500mg daily for 8 weeks was effective in lowering SU compared to placebo in healthy adults (mean reduction in serum urate was 0.35 mg/dL [95%CI 0.03-0.66], *p*-value 0.032) [1]. A similar result was reported in an updated meta-analysis [2]. Our study participants have a higher risk in developing gout and gout-related complications. Finding a potential low-cost, safe, readily available, and effective agent to reduce serum urate in a high-risk population, even when they have not manifested with gout or hyperuricemia, could be of interest to the community as well as the clinicians who are taking care of this population.

We, therefore, designed the study to test whether vitamin C 500mg twice daily could lower serum urate in participants without gout and/or hyperuricemia. Our description of these studies using the same dosage range (even in patients at high risk for gout) seem to us to as a sound basis for us selecting the same dose-even for individuals without hyperuricemia/gout with low little to no risk of harm.

How were the doses of vitamin C to be administered chosen?

**Response**: The basis for selecting that dose was grounded in existing studies using vitamin C for patients with gout/hyperuricemia. For example, a previous interventional study tested the efficacy of vitamin C 500mg once daily either with allopurinol or as a single agent versus allopurinol with dose titration. Although the efficacy of vitamin C 500mg once daily was significantly less efficacious compared to allopurinol, the magnitude of serum urate reduction as a single agent (0.23 mg/dL reduction) or combination with allopurinol (0.5 mg/dL) was similar to the meta-analysis mentioned above. The mild reduction in serum urate may be due to insufficient dose of vitamin C.

Vitamin C appears to exhibit linear kinetics at a lower dose and becomes non-linear at a higher dose to the saturable active transport mechanism in the absorption, distribution, and excretion. The maximum steady-state plasma concentration of ascorbic acid reaches about 70-80 uM [3]. The selection of vitamin C 500mg twice daily was to maintain steady-state concentration of ascorbic acid below the maximum concentration. Based on the pharmacokinetics of vitamin C (clearance of 6.02 L/hr and assuming the oral bioavailability of 1) [4], the estimated steady-state concentration of vitamin C 500mg is about 40 uM. Therefore, we choose a dose that was twice of the previous tested dose as the interventional dose but also one that is consistent with other previously conducted studies.

In order to communicate this in the article, we have added as requested above in the introduction, a brief description of an previous report that utilized 1000mg daily of vitamin C (reference 39) as well as the following statement at the end of the paragraph…in the introduction that reads…” Based on these studies, we selected a 500mg twice daily dose of vitamin C for our study having several sources of evidence for efficacy for individuals with hyperuricemia/gout, while displaying a favorable tolerance or safety profile for those without hyperuricemia or gout.”

Among the primary objectives, the authors should also introduce placebo especially in the patient group.

**Response**: We understand reviewer’s suggestion on (randomizing) study participants into a treatment and a control arm so the effect of vitamin C can be determined more definitely. However, given this is a pilot study in an under-studied population with limited budget, we focused on whether vitamin C is safe and potentially efficacious in both Hmong participants with and without gout and/or hyperuricemia. Introducing a placebo arm will be considered in the future confirmatory study should a positive signal (in terms of urate levels and microbiome changes) be observed from this pilot.

In my opinion, when analysing the data, the authors should also take gender into account.

**Response**: We agree that gender will, in fact, be considered in the analysis as described in the Analysis Plan: “Absolute SU changes from baseline to week 8 will be calculated using multiple linear regression adjusting for patients’ characteristics including age, gender, BMI, renal function, and concomitant medications.”

Fig. 1 is actually a table

**Response**: We agree with the reviewer that Fig. 1 is a table. However, due to the complex formatting within the table and potential issues when uploading the cell-based table in Microsoft Word, it is more reassuring to save this table as a figure, so the format remains intact. We will work with the editor to clarify this problem.

As far as blood and urine tests are concerned, the authors want to study the urate-creatinine trend.

**Response**: Thank you for the suggestion. You are correct in that the urate-creatinine trend will be considered in the future analyses.

It would also be good to analyse markers of oxidative stress, such as ROS, or indices of biological oxidative damage such as malondialdehyde and isoprostanes.

**Response**: These are excellent suggestions and, in general, we could consider numerous and varied biomarkers of oxidative stress ROS, etc. Practically speaking, however, we have limitations of funding and study design limitations that challenge our ability to both reliably collect and analyze these measures. At this time, we feel that this pilot study would provide a starting place to consider a more comprehensive study design more carefully (without limitations due to Covid-19) in the future.

Vitamin C at high plasma concentrations may act as a pro-oxidant (1) Have the authors ever tested this effect or is there any data for the treatment they want to perform?

**Response**: Thank you for the question: We have not yet tested for the effect of Vitamin C 500mg twice daily on pro-oxidant effects.

(2) Any treatment with vitamin C in patients genetically predisposed to high uric acid levels should be performed for life. According to the authors, would this not lead to an increased oxidative risk in these subjects?

1) Julia Kaźmierczak-Barańska , Karolina Boguszewska, Angelika Adamus-Grabicka Bolesław T. Karwowski. Two Faces of Vitamin C—Antioxidative and Pro-Oxidative Agent. Nutrients 2020, 12, 1501; doi:10.3390/nu12051501

2) Ian D. Podmore, Helen R. Griffiths, Karl E. Herbert, Nalini Mistry, Pratibha Mistry & Joseph Lunec. Vitamin C exhibits pro-oxidant properties. NATURE | VOL 392 | 9 APRIL 1998

Response: Indeed, most pharmacotherapeutic interventions to manage patients with gout are considered as medications for the long term as this risk persists in the absence of significant lifestyle changes for such patients. (e.g. careful dietary selections, reduction in alcohol consumption, etc). As designed, this study attempts to link changes in the serum urate with the vitamin C intake over several weeks (not chronically) and examine the impact of vitamin C on the microbiome within and between individuals with gout and/or hyperuricemia. If we identify a favorable effect of Vitamin C on serum urate in our study population for a short course of treatment, and a link between the microbiome and urate as well, we would naturally pursue more carefully designed dose ranging studies which test for the “optimal” dosage for the positive effects of vitamin C and any untoward effects of longer term use (pro-oxidative effects for example)

**Reference cited in the Response**

1.	Juraschek SP, Miller ER, 3rd, Gelber AC. Effect of oral vitamin C supplementation on serum uric acid: a meta-analysis of randomized controlled trials. Arthritis care & research. 2011;63(9):1295-306. Epub 2011/06/15. doi: 10.1002/acr.20519. PubMed PMID: 21671418; PubMed Central PMCID: PMCPMC3169708.

2.	Liu XX, Wang XX, Cui LL. Association between Oral vitamin C supplementation and serum uric acid: A meta-analysis of randomized controlled trials. Complement Ther Med. 2021;60:102761. Epub 2021/07/20. doi: 10.1016/j.ctim.2021.102761. PubMed PMID: 34280483.

3.	Stephenson CM, Levin RD, Spector T, Lis CG. Phase I clinical trial to evaluate the safety, tolerability, and pharmacokinetics of high-dose intravenous ascorbic acid in patients with advanced cancer. Cancer Chemother Pharmacol. 2013;72(1):139-46. Epub 2013/05/15. doi: 10.1007/s00280-013-2179-9. PubMed PMID: 23670640; PubMed Central PMCID: PMCPMC3691494.

4.	Nielsen TK, Hojgaard M, Andersen JT, Poulsen HE, Lykkesfeldt J, Mikines KJ. Elimination of ascorbic acid after high-dose infusion in prostate cancer patients: a pharmacokinetic evaluation. Basic Clin Pharmacol Toxicol. 2015;116(4):343-8. Epub 2014/09/16. doi: 10.1111/bcpt.12323. PubMed PMID: 25220574.
