# Supplementary material for: Hmong microbiome ANd Gout, Obesity, Vitamin C (HMANGO-C): A phase II clinical study protocol
Source: PLoS One. 2023 Feb 1;18(2):e0279830. doi: 10.1371/journal.pone.0279830 (PMC9891498; doi:10.1371/journal.pone.0279830)
Supplement: Attachment — Submitted filename: Second Response to Reviewers_11.09.2022.docx [file pone.0279830.s012.docx]

October 28, 2022

Re: Response to reviewer’s comments. (Manuscript ID PONE-D-22-04805R1)

Dear Dr. Vassalle and Reviewer,

Thank you for your feedback concerning our comprehensive response to the initial review. We are pleased that each of the reviewers took time to evaluate our responses and for the most part appreciated our responses as honest attempts to address their valued feedback. We are also happy to respond to additional comments made by the Reviewer 2.

Specific to Reviewer 2, we respectfully disagree with reviewer’s feedback on “most of the comments made were not taken into account”. Instead, we believe that most of the comments from Reviewer 2 (see **Comments 1-6** in the section of ***Reviewer 2 Comment from First Revision***) had been adequately addressed and incorporated into manuscript since Reviewer 2 did not have further suggestions on these comments.

Regarding reviewer 2’s suggestion on testing biomarkers of oxidative stress or DNA damage such as 8-oxoguanine and 8-oxoadenine (Kaźmierczak-Barańska *et al.* and Podmore *et al.*) due to increased oxidative risk from high dose vitamin C (500 mg twice daily), we have the following rebuttal. As previously described in the paper, the aim of our study is to evaluate the efficacy (by measuring serum urate change) and safety (by collecting patient reported adverse events) of ***vitamin C over the course of 8 weeks*** (not chronically) on the microbiome within and between individuals with and without gout and/or hyperuricemia.

The two key points here are (1) that we are looking for a signal from 8 weeks of vitamin C exposure (not chronic therapy as the reviewer suggested) and that (2) the dose chosen (1g/day) was by no means excessive, based on a variety of studies conducted in a variety of subjects using, in some cases much higher doses over similar, and in some cases, longer timeframes than our 8 weeks. Clinical studies have demonstrated the safety profile of a wide dose ranges of vitamin C in various populations (Yanase et al. *Pediatr Crit Care Med*. 2021;22(6):561-571; Andrés et al. *Cochrane Database Syst Rev*. 2021;11(11):CD010156.). Therefore, we are confident that the dose tested in the study is safe for the study participants in our setting. In addition, as for the suggestion to incorporate testing of biomarkers of oxidative stress or DNA damage in our study, we feel as stated before that the need for this is outside the scope of our study’s objectives. Including additional biomarkers would require a protocol change, revisions of informed consent, and the need to re-contact study participants which is not feasible given the study timeline of this study and resources. To reiterate our previous response, if we identify a favorable effect of Vitamin C on serum urate in our study population for our (8 week) short course of treatment, and a link between the microbiome and urate as well, we would naturally pursue more carefully designed dose ranging studies which would specifically test for the “optimal” dosages for the positive effects of vitamin C and any possible untoward effects (including pro-oxidative effects for example) especially if we pursued longer term (>8 week) use. It would also evaluate whether indeed, chronic dosing of vitamin C would be necessary or episodic (non-chronic use) of vitamin C would be more favorable. Since these are all possibilities it seems as if we should await a signal before planning the next series of studies.

We do note that Reviewer 2’s comments represent a statement of “In my opinion” and generally agree with the potential value in further study, but simply do not feel that Reviewer 2’s opinion justifies a change in our protocol at this stage, in order to investigate the possibility of a signal for the objective we are seeking. I will note that this is simply a pilot study seeking preliminary data in support of future more extensively designed investigations.

***Reviewer 2 Comment from Second Revision***

Most of the comments made were not taken into account. The authors state that they have difficulties in introducing other markers or the placebo due to their limited budget.

In my opinion, there are aspects not to be underestimated in this study, such as the production of oxygen free radicals in patients subjected to high doses of vitamin C for long periods.

*As a result of our careful consideration of “Reviewer 2’s Comments” we believe we have addressed them as above in a manner that does not require changes to the current version of the manuscript posted. This is why you will not see an updated version of the manuscript but simply our rebuttal to Reviewer 2’s comments as above.*

***Reviewer 2 Comment from First Revision***

This study protocol is well structured and well elaborated., although there are some critical points that the authors should remedy.

1. The authors should better explain the rationale for the administration of vitamin C to subjects without gout/hyperuricaemia.

**Response**: As described in the background, a previous meta-analysis had demonstrated at a median dose of vitamin C 500mg daily for 8 weeks was effective in lowering SU compared to placebo in healthy adults (mean reduction in serum urate was 0.35 mg/dL [95%CI 0.03-0.66], *p*-value 0.032) [1]. A similar result was reported in an updated meta-analysis [2]. Our study participants have a higher risk in developing gout and gout-related complications. Finding a potential low-cost, safe, readily available, and effective agent to reduce serum urate in a high-risk population, even when they have not manifested with gout or hyperuricemia, could be of interest to the community as well as the clinicians who are taking care of this population.

We, therefore, designed the study to test whether vitamin C 500mg twice daily could lower serum urate in participants without gout and/or hyperuricemia. Our description of these studies using the same dosage range (even in patients at high risk for gout) seem to us to as a sound basis for us selecting the same dose-even for individuals without hyperuricemia/gout with low little to no risk of harm.

2. How were the doses of vitamin C to be administered chosen?

**Response**: The basis for selecting that dose was grounded in existing studies using vitamin C for patients with gout/hyperuricemia. For example, a previous interventional study tested the efficacy of vitamin C 500mg once daily either with allopurinol or as a single agent versus allopurinol with dose titration. Although the efficacy of vitamin C 500mg once daily was significantly less efficacious compared to allopurinol, the magnitude of serum urate reduction as a single agent (0.23 mg/dL reduction) or combination with allopurinol (0.5 mg/dL) was similar to the meta-analysis mentioned above. The mild reduction in serum urate may be due to insufficient dose of vitamin C.

Vitamin C appears to exhibit linear kinetics at a lower dose and becomes non-linear at a higher dose to the saturable active transport mechanism in the absorption, distribution, and excretion. The maximum steady-state plasma concentration of ascorbic acid reaches about 70-80 uM [3]. The selection of vitamin C 500mg twice daily was to maintain steady-state concentration of ascorbic acid below the maximum concentration. Based on the pharmacokinetics of vitamin C (clearance of 6.02 L/hr and assuming the oral bioavailability of 1) [4], the estimated steady-state concentration of vitamin C 500mg is about 40 uM. Therefore, we choose a dose that was twice of the previous tested dose as the interventional dose but also one that is consistent with other previously conducted studies.

In order to communicate this in the article, we have added as requested above in the introduction, a brief description of an previous report that utilized 1000mg daily of vitamin C (reference 39) as well as the following statement at the end of the paragraph…in the introduction that reads…” Based on these studies, we selected a 500mg twice daily dose of vitamin C for our study having several sources of evidence for efficacy for individuals with hyperuricemia/gout, while displaying a favorable tolerance or safety profile for those without hyperuricemia or gout.”

3. Among the primary objectives, the authors should also introduce placebo especially in the patient group.

**Response**: We understand reviewer’s suggestion on (randomizing) study participants into a treatment and a control arm so the effect of vitamin C can be determined more definitely. However, given this is a pilot study in an under-studied population with limited budget, we focused on whether vitamin C is safe and potentially efficacious in both Hmong participants with and without gout and/or hyperuricemia. Introducing a placebo arm will be considered in the future confirmatory study should a positive signal (in terms of urate levels and microbiome changes) be observed from this pilot.

4. In my opinion, when analysing the data, the authors should also take gender into account.

**Response**: We agree that gender will, in fact, be considered in the analysis as described in the Analysis Plan: “Absolute SU changes from baseline to week 8 will be calculated using multiple linear regression adjusting for patients’ characteristics including age, gender, BMI, renal function, and concomitant medications.”

5. Fig. 1 is actually a table

**Response**: We agree with the reviewer that Fig. 1 is a table. However, due to the complex formatting within the table and potential issues when uploading the cell-based table in Microsoft Word, it is more reassuring to save this table as a figure, so the format remains intact. We will work with the editor to clarify this problem.

6. As far as blood and urine tests are concerned, the authors want to study the urate-creatinine trend.

**Response**: Thank you for the suggestion. You are correct in that the urate-creatinine trend will be considered in the future analyses.

7. It would also be good to analyse markers of oxidative stress, such as ROS, or indices of biological oxidative damage such as malondialdehyde and isoprostanes.

**Response**: These are excellent suggestions and, in general, we could consider numerous and varied biomarkers of oxidative stress ROS, etc. Practically speaking, however, we have limitations of funding and study design limitations that challenge our ability to both reliably collect and analyze these measures. At this time, we feel that this pilot study would provide a starting place to consider a more comprehensive study design more carefully (without limitations due to Covid-19) in the future.

8. Vitamin C at high plasma concentrations may act as a pro-oxidant (1) Have the authors ever tested this effect or is there any data for the treatment they want to perform?

**Response**: Thank you for the question: We have not yet tested for the effect of Vitamin C 500mg twice daily on pro-oxidant effects.

(2) Any treatment with vitamin C in patients genetically predisposed to high uric acid levels should be performed for life. According to the authors, would this not lead to an increased oxidative risk in these subjects?

1) Julia Kaźmierczak-Barańska , Karolina Boguszewska, Angelika Adamus-Grabicka Bolesław T. Karwowski. Two Faces of Vitamin C—Antioxidative and Pro-Oxidative Agent. Nutrients 2020, 12, 1501; doi:10.3390/nu12051501

2) Ian D. Podmore, Helen R. Griffiths, Karl E. Herbert, Nalini Mistry, Pratibha Mistry & Joseph Lunec. Vitamin C exhibits pro-oxidant properties. NATURE | VOL 392 | 9 APRIL 1998

Response: Indeed, most pharmacotherapeutic interventions to manage patients with gout are considered as medications for the long term as this risk persists in the absence of significant lifestyle changes for such patients. (e.g., careful dietary selections, reduction in alcohol consumption, etc). As designed, this study attempts to link changes in the serum urate with the vitamin C intake over several weeks (not chronically) and examine the impact of vitamin C on the microbiome within and between individuals with gout and/or hyperuricemia. If we identify a favorable effect of Vitamin C on serum urate in our study population for a short course of treatment, and a link between the microbiome and urate as well, we would naturally pursue more carefully designed dose ranging studies which test for the “optimal” dosage for the positive effects of vitamin C and any untoward effects of longer term use (pro-oxidative effects for example)
